# Zinc-MOF-carbon hollow sphere hybrid nanozyme for highly selective colorimetric detection of mercury ions

**DOI:** 10.1039/d6ra02355j

**Published:** 2026-08-06

**Authors:** Garima, Kushal Arya, Indu Sharma, Ennio Zangrando, Vinod Kumar, Rakesh Kumar Bhardwaj, Ramesh Kataria

**Affiliations:** a Department of Chemistry and Centre of Advanced Studies in Chemistry, Panjab University Chandigarh 160014 India rkataria@pu.ac.in; b University Center for Research and Development, Chandigarh University Mohali Punjab 140301 India; c Department of Chemical and Pharmaceutical Sciences, University of Trieste Trieste 34127 Italy; d Department of Chemistry, Central University of Haryana Mahendragarh 123029 India

## Abstract

Mercury pollution poses significant environmental and health hazards, necessitating rapid, specific, and sensitive detection methods. Here, a novel zinc-based metal–organic framework (PUC-15) was synthesized, followed by the development of a hybrid nanozyme composite (PUC-15@MCHS) by incorporating the PUC-15 into mesoporous carbon hollow spheres, designed for the effective colorimetric detection of mercury ions (Hg^2+^). The synthesized PUC-15 and PUC-15@MCHS materials were fully characterized using SCXRD, PXRD, FTIR, SEM, TEM, BET, *etc.* The crystal structure of PUC-15 shows a three-fold interpenetrated network with Zn(ii) displaying a distorted tetrahedral coordination environment built by a nitrogen donor from a bridging dipyridyl ligand and three oxygen atoms from two monodentate L_1_ carboxylates (L_1_ = 5,5′-(1,3,6,8-tetraoxo-1,3,6,8-tetrahydrobenzo[*lmn*]-3,8-phenanthro-line-2,7-diyl)diisophthalic acid) and one acetate group, respectively. The PUC-15@MCHS acts as a nanozyme that exhibits vigorous peroxidase-mimicking activity, efficiently catalyzing the oxidation of 3,3′,5,5′-tetramethylbenzidine (TMB) in the presence of hydrogen peroxide (H_2_O_2_), resulting in a blue oxidized product detectable at 652 nm. Kinetic studies revealed a low Michaelis-Mentan constant (*K*_m_) for H_2_O_2_ (0.27 mM), indicating high substrate affinity and catalytic efficiency. Zeta potential analysis showed a negative surface charge (−18.6 mV), which enhances electrostatic attraction toward Hg^2+^ ions. XPS confirmed the specific binding interactions between mercury ions and nitrogen/oxygen-containing active sites on the composite surface. The developed sensor exhibited excellent selectivity toward Hg^2+^ in the presence of competing metal ions, with a LOD of 0.59 µM and a good linear response (*R*^2^ = 0.978), highlighting PUC-15@MCHS as an efficient and reliable nanozyme for mercury monitoring in environmental samples.

## Abstract 2

Mercury pollution poses significant environmental and health hazards, necessitating rapid, specific, and sensitive detection methods.

## Graphical abstract

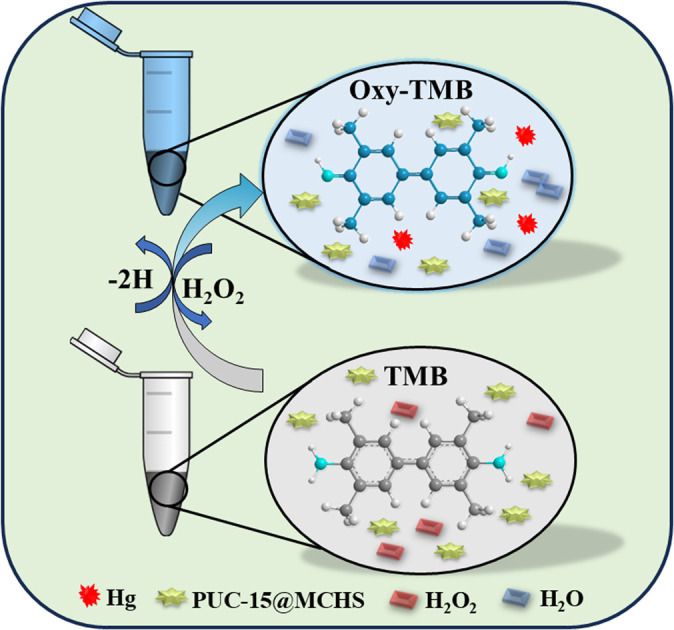

## 1 Introduction

The continuous release of mercury into the atmosphere constitutes a critical environmental concern across global platforms, arising from both natural sources and human activities.^1^ The gradual build-up of trace amounts of organic mercury through the food chain can lead to severe neurotoxic effects and potentially harm multiple organs in the human body.^2^ To mitigate the adverse impacts of mercury contamination on human health and ecosystems, numerous detection methods, including instrumental, electrochemical approaches and optical techniques, have been developed to track Hg^2+^ in aqueous environments and evaluate associated risks.^3–6^ Among these approaches, nanomaterials have drawn considerable attention due to their notable optical, electrical and mechanical properties.^7–10^ Bio-enzymes also represent an important category of nanomaterials, but face limitations such as laborious preparation procedures, susceptibility to denaturation, high manufacturing costs and difficulties in recycling.^11^ To address these limitations, considerable effort has been directed toward the development of nanozyme materials that exhibit enzyme-like catalytic activity.^12^ Nanozymes have been widely explored for biosensing and enzymatic assays because of their structural stability and adjustable catalytic properties.^13^ Among the different types of nanozymes reported so far, peroxidase-mimicking systems were the first to be identified and remain the most extensively investigated.^14^ Owing to their vivid and easily observable colorimetric responses, peroxidase (POD)-mimicking materials have demonstrated potential in detecting metal ions,^15^ glutathione,^16,17^ ascorbic acid,^18^ antibacterial applications,^19^ catalytic applications,^20^ and glucose sensing.^21,22^ In comparison with conventional analytical methods, nanozyme-based sensing utilizes controllable catalytic color changes triggered by Hg^2+^ ions, offering highly sensitive, selective, and interference-resistant detection platforms suitable for complex matrices. These techniques offer high selectivity, sensitivity and excellent resistance to interference, making them suitable for detecting Hg^2+^ in complex environments.^23,24^

In recent years, metal–organic frameworks (MOFs) have gained recognition as a versatile class of nanozymes, valued for their unique porosity, tunable structures, and catalytic capabilities.^25^ MOFs consist of metal ions or clusters linked by organic ligands, forming highly porous crystalline networks. The modular nature of MOFs enables them to function as biomimetic catalysts, where the metal nodes act as functional analogues of metalloenzyme active sites.^26,27^ For instance, a porphyrin-based MOF composite AuNPsPCN-224(Fe) acts as a nanozyme and enables sensitive catalysis of glucose to generate H_2_O_2_ in the presence of oxygen.^28^ Similarly, Ce-MOF has demonstrated enzyme-mimicking activity by effectively catalyzing the dephosphorylation of phosphopeptides, in which its Ce(iii) node closely resembles the active sites of phosphate.^29^ In a similar vein, a bismuth-based MOF nanozyme (BiO-BDC-NH_2_) was developed for colorimetric and electrochemical detection of chromium.^30^ The exceptionally high surface area and porous frameworks of MOFs allow substrates to diffuse easily and reach catalytic spots, while confined pore spaces improve selectivity. These characteristics contribute to their growing applicability in biosensing, environmental remediation and diagnostic platforms. Nevertheless, the practical deployment of MOFs is often constrained by inherent drawbacks such as poor mechanical robustness, low electrical conductivity, and susceptibility to degradation under moisture and light exposure. To address these limitations in MOFs, researchers have extensively investigated their combination with diverse carbonaceous materials, including carbon nanotubes, graphene, carbon nanofibers, activated carbon, carbon black, and carbon quantum dots.^31–36^ The resulting MOF-carbon composites benefit from synergistic interactions between the crystalline porous frameworks and conductive carbon matrices, leading to enhanced structural stability, improved charge transport, and greater chemical durability. Expanding on this, mesoporous carbon hollow spheres (MCHS) have emerged as attractive alternatives for their hollow structures, high surface areas, and ordered mesoporosity.^37–39^ MCHS composites further enhance electron transport and active site utilization, for instance, such as Pt-MCHS systems that exhibit high durability and activity in oxygen reduction reactions due to efficient electronic coupling and structural support.^40–49^ However, the incorporation of MOF@MCHS hybrid material is not well explored, as only a few studies have demonstrated improved charge mobility, electrolyte accessibility, and structural stability, highlighting their potential for advanced optical sensors.^50^

In this work, a hybrid nanozyme (PUC-15@MCHS) has been synthesized by integrating a novel zinc-based MOF (PUC-15) with MCHS and explored for selective and colorimetric detection of Hg^2+^ ions, utilizing peroxidase-mimicking activity. Notably, the sensor enables instantaneous and visually observable color change for naked-eye detection of Hg^2+^, highlighting its practical advantage over the pristine MOF and previously reported systems.

## 2 Experimental

### 2.1 Materials and methods

Chemicals employed in this study included the pre-synthesized ligand, 1,2-di(4-pyridyl)ethylene, obtained from Sigma-Aldrich, and 3,3′,5,5′-tetramethylbenzidine, procured from Avra Synthesis. Mercuric acetate was purchased from Sigma-Aldrich, Zn(NO_3_)_2_·4H_2_O from CDH Chemicals, and solvents including DMF, MeOH, and acetic acid from FINAR. Analytical-grade reagents were used and employed without prior purification.

FT-IR spectra were collected using a PerkinElmer FTIR 2000 spectrometer. Powder X-ray diffraction (PXRD) patterns were obtained on a PANalytical X'Pert Pro instrument utilizing Cu-Kα radiation (*λ* = 1.54059 Å). Thermogravimetric analysis (TGA) was conducted on an SDT Q600 (V20.9 Build 20), with samples heated at 10 °C min^−1^ under a nitrogen atmosphere. Surface morphology was examined using high-resolution transmission electron microscopy (HR-TEM, Tecnai F-20) and field-emission scanning electron microscopy (FESEM, Hitachi SU8010). Zeta potential measurements and X-ray photoelectron spectroscopy (XPS) analyses were conducted employing a PHI 5000 VersaProbe III and a ThermoFisher Nexsa instrument, respectively. Nitrogen adsorption–desorption isotherms and Brunauer–Emmett–Teller (BET) surface area analyses were performed using an Autosorb IQ-XR-XR-XR (3 Stat.) equipped with Viton seals. UV-visible absorption spectra were recorded on a K-LAB Alpha UV-visible spectrophotometer. Diffraction data of the compound were collected at room temperature using a Rigaku SuperNova diffractometer equipped with a microfocus sealed X-ray tube and Mo-Kα radiation (*λ* = 0.71073 Å), and a CCD HyPix3000 detector. The CrysAlisPro software^51^ was used for data acquisition and reduction. The absorption was corrected by the SCALE3 ABSPACK multi-scan method.^52^ The structure was solved with SHELXT^53^ and refined by the full-matrix least-squares methods based on *F*^2^ implemented in ShelXL^54^ with anisotropic non-H atoms. Hydrogen atoms were fixed at geometrically calculated positions. Data were treated with the Squeeze program^55^ to take into account disordered solvent molecules (potential solvent volume = 917.1 Å^3^, 32.8% of the unit cell). Some selected bond length data are provided in Table S1. Crystallographic data of PUC-15 have been deposited with the Cambridge Crystallographic Data Centre (CCDC) under the CCDC number 2497354. A copy of the data can be obtained by applying to the CCDC, 12 Union Road, Cambridge CB2 1EZ, U.K. (fax: +44 (1223) 336 033; email: https://www.deposit@ccdc.cam.ac.uk), without any cost.

#### 2.1.1 Crystallographic data

C_23_H_13_N_2_O_8_Zn, *M* = 510.72, monoclinic, space group *P*2_1_/*c*, *a* = 18.4934(8) Å, *b* = 9.2191(4) Å, *c* = 16.3977(7) Å, *β* = 92.741(4)°, *V* = 2792.5(2) Å^3^, *Z* = 4, *D*_c_ = 1.215 g cm^−3^, µ(Mo-Kα) = 0.921 mm^−1^, *F*(000) = 1036, *θ* range = 3.24 to 27.18°. Final *R*_1_ = 0.0470, *wR*_2_ = = 0.1138, *S* = 1.058 for 308 parameters and 11 433 reflections, 5608 unique [*R*_int_ = 0.0697], of which 3714 with *I* > 2*σ*(*I*), max positive and negative peaks in Δ*F* map 0.396, −0.292e. Å^−3^.

### 2.2 Synthesis of PUC-15 and PUC-15@MCHS

#### 2.2.1 Synthesis of PUC-15

**Fig. 1:**
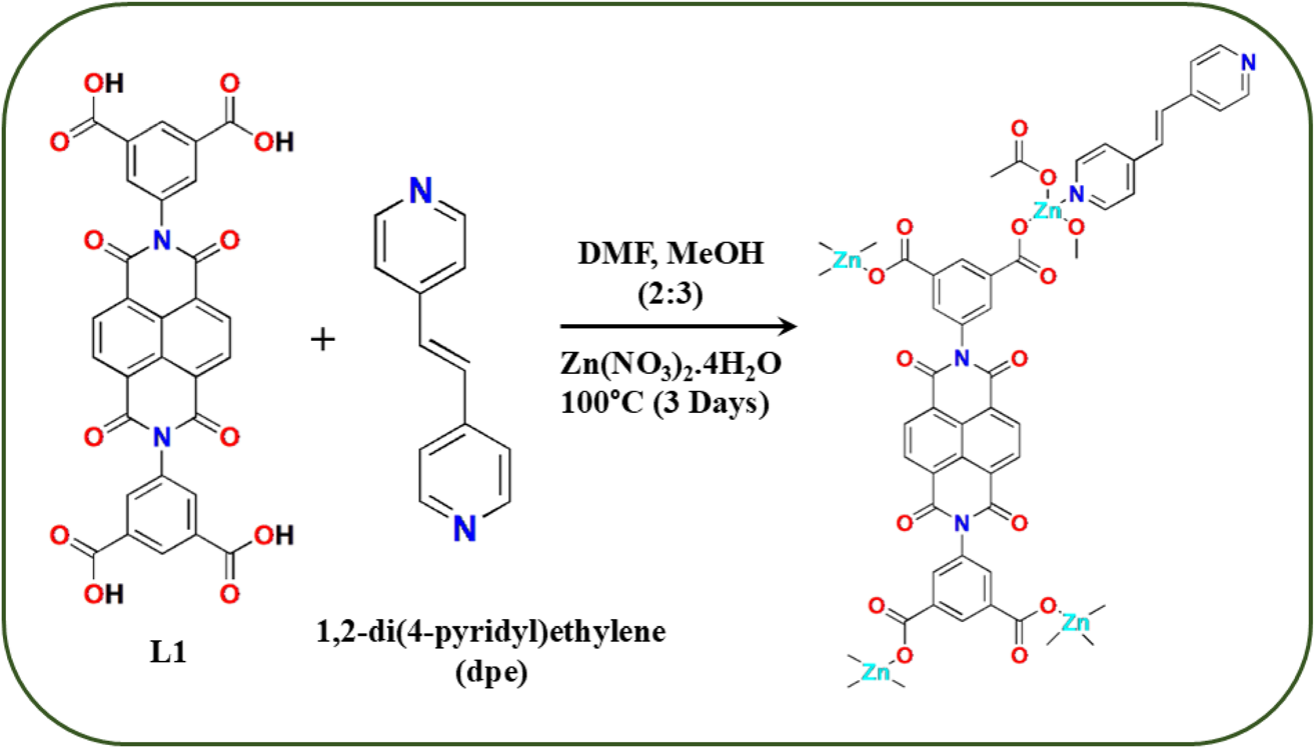
Schematic representation of the synthesis of PUC-15.

PUC-15 was synthesized *via* a solvothermal method using a mixture of ligand L_1_ (L_1_ = 5,5′-(1,3,6,8-tetraoxo-1,3,6,8-tetrahydrobenzo[*lmn*]-3,8-phenanthroline-2,7-diyl)diisophthalic acid), 1,2-di(4-pyridyl)ethylene (dpe), and zinc nitrate tetrahydrate Zn(NO_3_)_2_·4H_2_O, which were combined in a molar ratio of 1 : 1 : 2, respectively. The reactants were dissolved in 3 mL of a DMF–methanol solvent mixture (2 : 3 v/v). The resulting solution was then stirred at 350 rpm for 10 minutes, during which a few drops of acetic acid (AcOH) were added to regulate crystallization. The solution was then heated solvothermally at 100 °C for 48 hours. The yield was calculated to be 84% based on 1,2-di(4-pyridyl)ethylene. The synthetic scheme of the MOF is illustrated in Fig. 1.

#### 2.2.2 Synthesis of PUC-15@MCHS composite

**Fig. 2:**
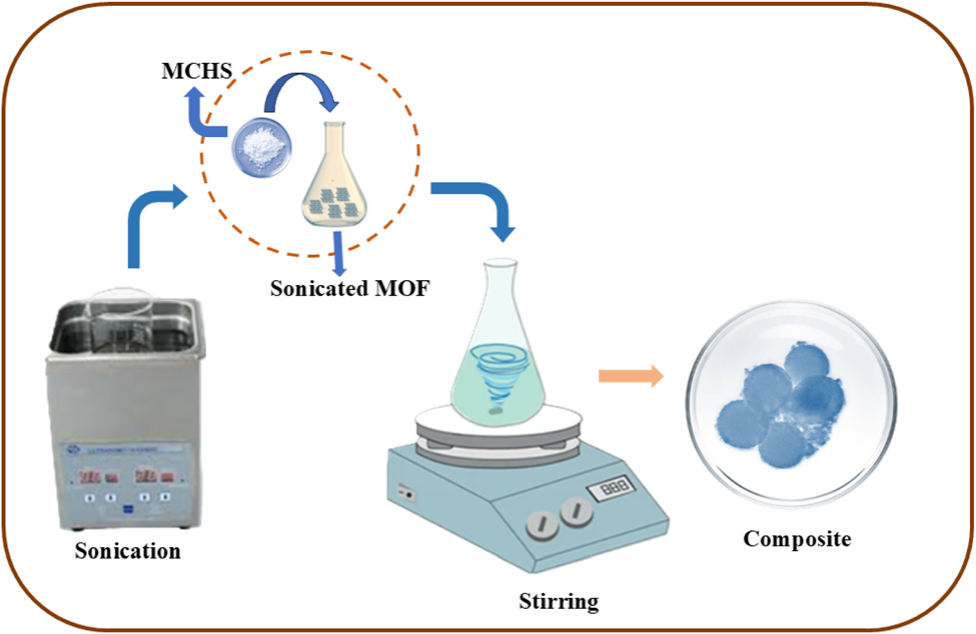
Illustration of the key steps of the composite synthesis process.

Mesoporous carbon hollow spheres (MCHS) were synthesized according to a previously reported procedure.^56,57^ To fabricate the PUC-15@MCHS composite, the as-synthesized PUC-15 crystals were ultrasonicated in double-distilled water for 1 h to achieve uniform dispersion. Subsequently, the pre-synthesized MCHS were added to the MOF dispersion under continuous stirring. To ensure intimate interfacial interaction between MOF and carbon spheres, the mixture was stirred overnight at room temperature, resulting in the successful formation of the composite. Centrifugation was used to collect the resulting material, which was then repeatedly washed with ethanol and deionized water before vacuum drying. The schematic representation of the composite fabrication process is shown in Fig. 2, which depicts the integration of MCHS into the MOF structure.

## 3 Results and discussion

### 3.1 Structural description of PUC-15

**Fig. 3:**
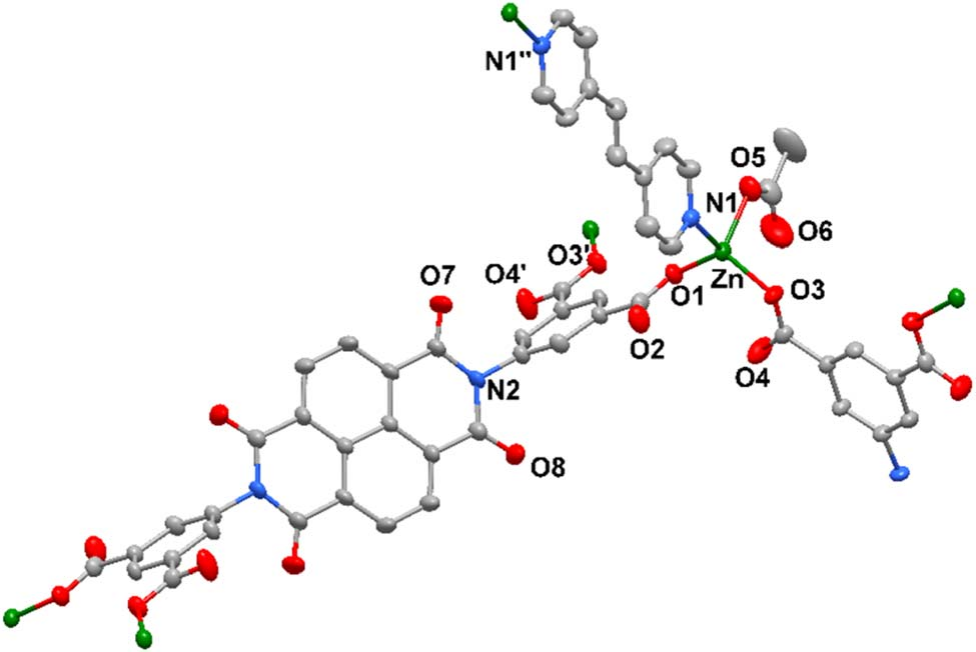
ORTEP view of the coordination sphere of Zn atom and ligand connections (H atoms omitted for clarity). Symmetry codes: (′) at 1 − *x*, 2 − *y*, 2 − *z*, (″) *x*, 0.5 − *y*, −0.5 + *z*.

The structure of compound [Zn_2_(Ac)_2_(dpe)(L_1_)]_*n*_ consists of three-fold interpenetrated networks of topology with point symbol {3^6^.4^2^.5^10^.6^3^}. Fig. 3 shows an ORTEP view with the atom-numbering scheme of the metal coordination geometry about the Zn ion, and Table S2 reports a selection of bond distances and angles. The tetradeprotonated L_1_ acts as a µ_4_ ligand, dpe behaves as a bridging species, and both are located on an inversion centre, so that half of them is crystallographically independent.

The Zn ion exhibits a distorted tetrahedral coordination geometry built by the nitrogen donor of the bridging dipyridyl molecule and by three carboxylate oxygen atoms, from two symmetry-related monodentate L_1_ carboxylates and from an acetate anion. The Zn–O bond lengths are comparable between 1.956(2)–1.9729(19) Å, while the Zn–N bond is slightly longer, at 2.079(2) Å. These values are in agreement with those detected in two Zn polymeric structures with carboxylate and N donor ligands and in the discrete neutral complex [Zn(Ac)_2_(Bzm)_2_] (Bzm = benzoimidazol derivative).^58–60^

**Fig. 4:**
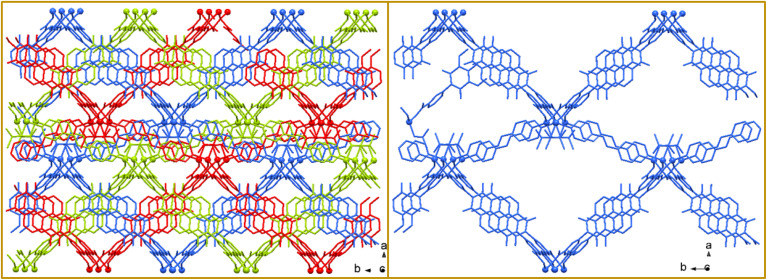
(a) The three interpenetrated nets and, on the right, of a single net of compound [Zn_2_(Ac)_2_(dpe)(L_1_)]_*n*_ viewed down axis-*c*.

**Fig. 5:**
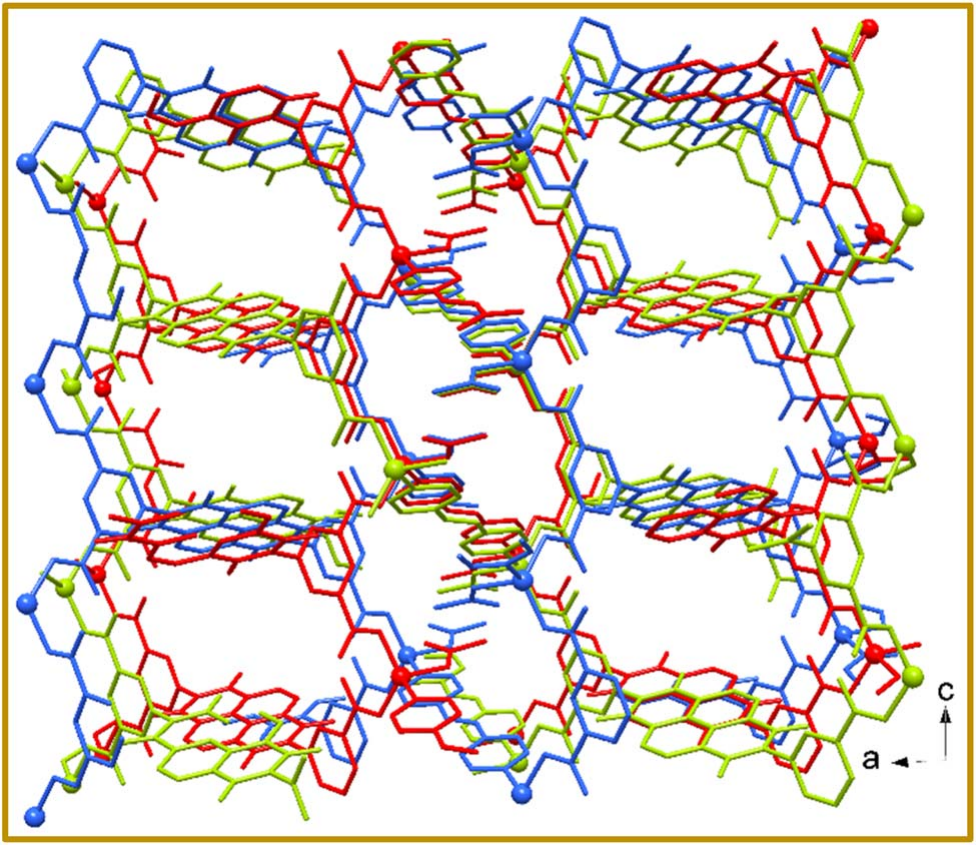
Perspective view down axis-*b* showing the channels in the crystal packing of [Zn_2_(Ac)_2_(dpe)(L_1_)]_*n*_.

Fig. 4 shows a view of the three interpenetrated nets of the compound viewed down axis-*c*, and Fig. 5 illustrates the channels in the crystal packing which account for 917.1 Å^3^, 32.8% of the unit cell volume. Assuming a volume of 35–40 Å^3^ for a water molecule, the void in the cell might accommodate *ca.* 24 water molecules, 6 per Zn unit. In the network, the metal atoms are spaced by 13.58(1) Å along the rigid dpe, by 19.79(1), 21.43(1), 21.56(1) Å between opposite carboxylate groups of L_1_, and by 8.39 Å between the coordinating carboxylate oxygen of the same isophthalate fragment (O1 and O3′ in Fig. 3).

### 3.2 FTIR, PXRD, TGA and BET analysis of PUC-15 and PUC-15@MCHS

**Fig. 6:**
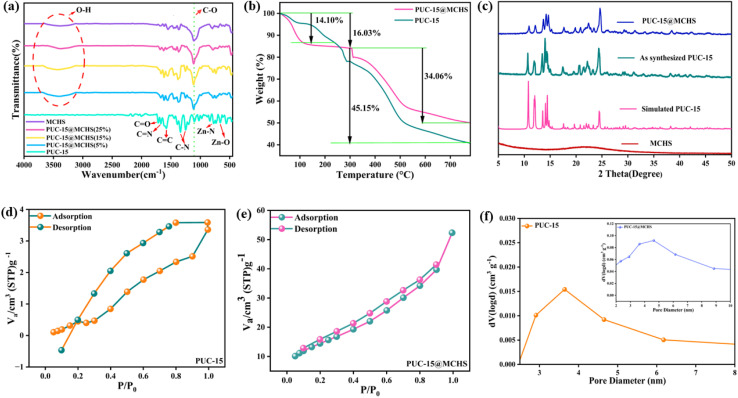
FT-IR spectra (a); TGA profiles (b); PXRD patterns (c); nitrogen adsorption–desorption isotherms of PUC-15 MOF (d) and PUC-15@MCHS composite (e); pore size distribution and volume analysis of PUC-15 (f) with inset showing corresponding data for the composite.

FTIR spectra of PUC-15 MOF, MCHS and PUC-15@MCHS composites of different compositions (5, 15, 25 wt%) are shown in Fig. 6a. The spectrum of PUC-15 MOF exhibits characteristic peaks at 1717 and 1668 cm^−1^, corresponding to C=O and C=N stretching vibrations, respectively.^61^ The band observed at 1580 cm^−1^ is attributed to C=C stretching, reflecting the presence of aromatic moieties.^62^ Signals at 1335 and 1246 cm^−1^ are due to C–N vibrations, and bands near 1094 and 1024 cm^−1^ correspond to C–N or C–O stretching. Out-of-plane C–H bending is observed at 833, 769, and 714 cm^−1^.^63^ Additionally, peaks at 770 and 664 cm^−1^ confirm the presence of Zn–N and Zn–O bonding, supporting the coordination of zinc within the framework and the successful synthesis of MOF.^64^ The broad absorption band near 3370 cm^−1^ is assigned to the O–H stretching mode, corresponding to surface hydroxyl groups of MCHS, which persists across all PUC-15@MCHS compositions, with peak intensities increasing proportionally with MCHS weight percentage. All these observations indicate successful composite formation without any structural disruption in the MOF structure.

Thermogravimetric analysis under N_2_ atmosphere was carried out to evaluate the thermal behavior of pristine PUC-15 and PUC-15@MCHS (Fig. 6b). For clarity, the main manuscript presents the thermogram of a representative PUC-15@MCHS composite, while the full composition-dependent comparison is provided in the SI. Pristine PUC-15 exhibits an initial low-temperature weight loss attributed to the removal of adsorbed or entrapped solvent molecules, followed by further mass loss at elevated temperature due to gradual framework decomposition. As shown in Fig. 6b, PUC-15@MCHS retains a higher residual mass than pristine PUC-15, reflecting the contribution of thermally persistent carbonaceous MCHS in the hybrid material. In addition, the EDS and TGA results of the full composition series indicate a consistent compositional trend with increasing MCHS content. These observations further support the successful incorporation of MCHS into the hybrid system. The comparative EDS results are summarized in Table S3 and Fig. S1, while the TGA curves of all compositions are presented in Fig. S2.

In order to check the crystallinity and phase purity of PUC-15 and its MCHS-based composite, PXRD studies were carried out. The PUC-15 sample displayed sharp and well-defined peaks at 2*θ* values of 10.8, 11.9, 13.6, 14.4, and 24.4°, which can be attributed to the (002), (102), (210), (012), (111), and (114) lattice planes. The experimental peaks closely resemble the simulated pattern of PUC-15, indicating the successful synthesis (Fig. 6c). The PXRD pattern of PUC-15@MCHS exhibits the characteristic diffraction peaks of PUC-15, confirming the preservation of the MOF framework after composite formation. Compared with pristine PUC-15, the diffraction peaks of the composite show reduced intensity and slight broadening, which can be attributed to the presence of the amorphous MCHS matrix and the partial reduction in crystallinity upon integration. The mesoporous properties of PUC-15 and PUC-15@MCHS were further investigated through nitrogen adsorption–desorption isotherm (BET). A typical Type II isotherm with a noticeable hysteresis loop was observed in the case of PUC-15, reflecting the presence of microporous features as shown in Fig. 6d. The calculated specific surface area was 8.36 m^2^ g^−1^, while pore size distribution analysis indicated micropores with sizes of approximately 3.64 nm. On the other hand, the PUC-15@MCHS composite demonstrated a Type IV isotherm featuring a H_3_ hysteresis loop, indicating mesoporous behavior introduced by the mesoporous carbon hollow spheres (Fig. 6e). This composite exhibited a significantly increased surface area of 84.18 m^2^ g^−1^ and an average pore size of about 4.64 nm (Fig. 6f). The synergistic interfacial effects and improved reaction kinetics make PUC-15@MCHS a better nanozyme option compared to bare MOF, delivering the substrate access and site exposure needed for detection.

### 3.3 FE-SEM and HR-TEM analysis

**Fig. 7:**
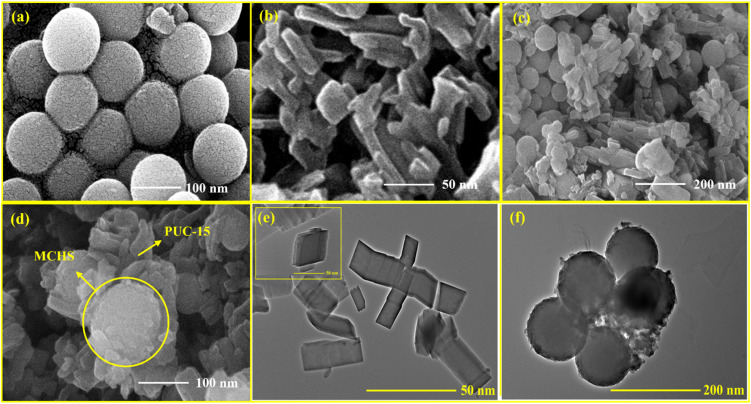
FESEM images showing the morphology of MCHS (a); PUC-15 MOF (b); and the PUC-15@MCHS composite (c and d); HRTEM images of PUC-15 (e) and the PUC-15@MCHS composite (f) illustrating structural integration at the nanoscale.

The surface morphologies of synthesized materials were investigated by using field emission scanning electron microscopy (FESEM). The morphology of mesoporous carbon holospheres (MCHS) displayed well-defined spherical particles with smooth surfaces and a uniform diameter of approximately 100 nm (Fig. 7a), which matches the reported literature confirming successful synthesis.^65^Fig. 7b shows the FESEM image of the pure PUC-15 MOF, revealing uniform, rod-shaped crystals with a smooth surface. In the PUC-15@MCHS composite, MOF rods appear embedded or anchored onto the MCHS surface, forming a heterogeneous architecture as depicted in Fig. 7c and d. The interfacial contact between MOF and MCHS shows successful hybridization of both components to form a composite. The composite PUC-15@MCHS formed with more exposed sites, enhanced surface area and structure stability, making it a promising material for sensing applications.

The HR-TEM images clearly illustrate that PUC-15 forms well-defined, block-shaped crystals with smooth surfaces (Fig. 7e). A closer examination of the PUC-15@MCHS composite through HR-TEM (Fig. 7f) shows the PUC-15 crystals randomly distributed across the MCHS surface. Further, the average size distribution of PUC-15@MCHS from the size histogram is found to be 56 nm (Fig. S3). Additional compositional analysis, including elemental mapping for all synthesized materials, is provided in the SI (Fig. S4–S8).

### 3.4 XPS analysis of PUC-15

**Fig. 8:**
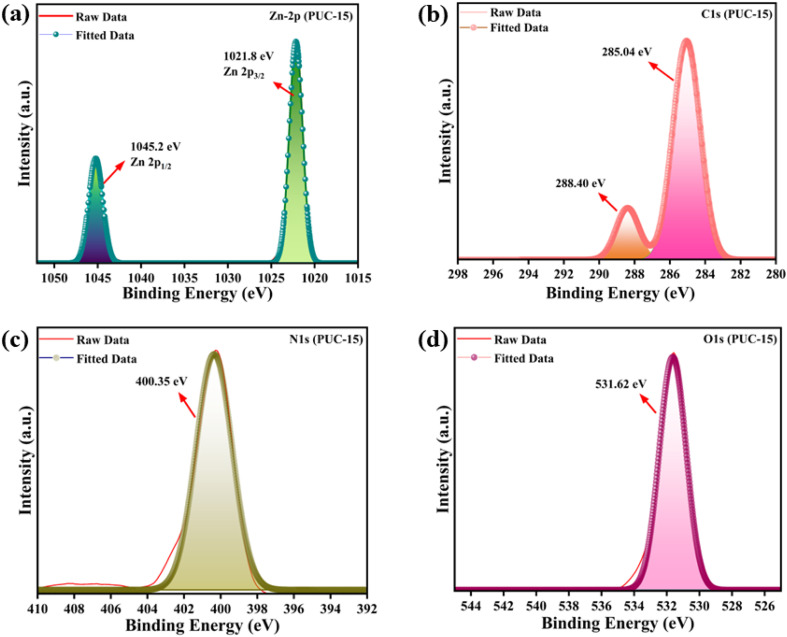
XPS spectra of PUC-15 MOF showing high-resolution scans for Zn 2p (a); C 1s (b); N 1s (c); and O 1s (d); confirming the elemental composition and chemical states.

XPS confirmed the chemical environment and coordination in PUC-15. The Zn 2p spectrum (Fig. 8a) shows two peaks at 1021.8 eV (Zn 2p_3/2_) and 1045.2 eV (Zn 2p_1/2_), characteristic of Zn^2+^ coordinated with carboxylate and pyridine ligands. The C 1s spectrum (Fig. 8b) exhibits peaks at 285.04 eV and 288.40 eV, corresponding to unsaturated (C=C, C=N) and saturated/oxidised (C–C, C–N, C=O) carbon environments, respectively. The N 1s signal at 400.35 eV (Fig. 8c) indicates the presence of nitrogen in aromatic rings, consistent with pyridine coordination. A single O 1s peak at 531.62 eV (Fig. 8d) confirms the presence of oxygen from carboxylate groups. These results verify the successful formation of the Zn(ii)-based MOF structure with coordinated carboxylate and pyridine linkers. In addition to XPS evidence for the chemical environment and metal–ligand coordination in pristine PUC-15, the surface composition of PUC-15@MCHS was also investigated. High-resolution XPS spectra of Zn 2p, C 1s, N 1s and O 1s are presented in Fig. S9–S13. A comparison of the binding energies of pristine PUC-15 and PUC-15@MCHS (Table S4) revealed slight shifts in the characteristic Zn 2p, C 1s, N 1s and O 1s peaks after composite formation. These shifts indicate changes in the local electronic environment of the constituent atoms, suggesting interfacial interaction between PUC-15 and MCHS.^66^ At the same time, the characteristic Zn–ligand coordination features of PUC-15 were retained, confirming preservation of the MOF framework following composite formation.

### 3.5 Peroxidase-like catalytic activity of nanozyme PUC-15@MCHS

The peroxidase-like behavior of the nanocomposite was assessed through a redox reaction-based system. TMB was used as a chromogenic substrate, which imparts a blue colour when it gets oxidised, showing its characteristic peak at 652 nm in UV-visible spectroscopic analysis. To run the reaction at optimum pH conditions, an acetate buffer was used as a medium. Nanozymatic action was evaluated based on the redox reaction occurring between the nanocomposite and Hg^2+^ in the presence of H_2_O_2,_ which acts as an oxidizing agent, whereas the nanocomposite behaves as an adsorbent providing the catalytic surface. To clarify the role of hydrogen peroxide, time-resolved studies have been carried out. The study was conducted for the system with and without H_2_O_2_, *i.e.*, (buffer/TMB/PUC-15@MCHS) and buffer/TMB/H_2_O_2_/PUC-15@MCHS. It is observed that there is no significant difference in the absorbance intensity for the system without H_2_O_2,_ but there is an apparent increase in absorbance for the system containing H_2_O_2_. The reaction system was incubated for a period of 30 min, and the results are consistent, as shown in Fig. (S14.a). Now, the role of the nanocomposite as a potent nanozyme was assessed. The reaction system shows very little absorbance intensity and negligible colour change in the absence of nanocomposite, indicating slow oxidation of TMB, but there is a remarkable change in the intensity and colour on addition of nanocomposite, confirming the nanozymatic activity of PUC-15@MCHS (Fig. S14.b). To elucidate the contribution of each component toward the catalytic performance, control experiments were carried out using bare MCHS, pristine PUC-15, a physically mixed PUC-15/MCHS sample, and the synthesized PUC-15@MCHS composite under identical conditions (Fig. S15). Bare MCHS exhibited negligible catalytic activity, whereas pristine PUC-15 showed a moderate response. The physically mixed sample displayed improved activity compared to the individual components but remained inferior to that of PUC-15@MCHS. The superior catalytic performance of PUC-15@MCHS indicates that the enhancement cannot be attributed solely to the presence of both components. Instead, the synthesis process promotes effective interfacial contact between PUC-15 and MCHS, leading to synergistic interactions that contribute to the observed catalytic activity. The catalytic activity comparison of different composite compositions is provided in Fig. S16, based on which PUC-15@MCHS (15%) was selected as the optimized composition for all subsequent nanozyme studies.

### 3.6 Time-dependent catalytic response

**Fig. 9:**
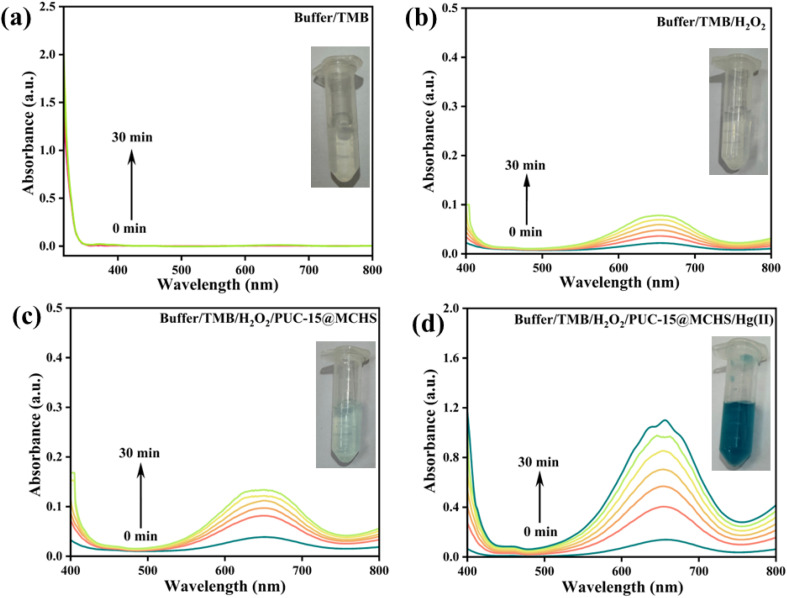
Time-dependent monitoring of the colorimetric reaction during nanozyme-mediated oxidation of TMB with buffer and TMB (a); addition of H_2_O_2_ (b); nanozyme catalyst (c); and final mixture containing mercury (d).

To explore the nanozymatic activity of the material, time studies for various reaction systems have been carried out to monitor the oxidation of TMB as a function of time. The time study was analyzed by sequential addition of reaction components at their respective optimal concentrations. Experiments were performed in acetate buffer medium at room temp. The absorbance of oxidized TMB was recorded at 652 nm at regular time intervals up to 30 min. Negligible colour development was observed before PUC-15@MCHS addition, even after 30 min, indicating minimal TMB oxidation in H_2_O_2_ alone, as shown in Fig. 9.

A subtle color shift occurred upon addition of the nanocomposite, whereas Hg^2+^ addition brought a rapid and pronounced colour change that can be readily detected by the naked eye, as shown in Fig. 9d inset. These results clearly demonstrate the role of Hg^2+^ as a catalyst to accelerate TMB oxidation. The colour intensity of the reaction steadily deepened over time, indicating the continuous progression of the oxidation process as more TMB is converted. Notably, even concentrations of Hg(ii) at the micromolar level proved adequate to trigger this colorimetric shift.

### 3.7 Optimization of catalytic conditions

**Fig. 10:**
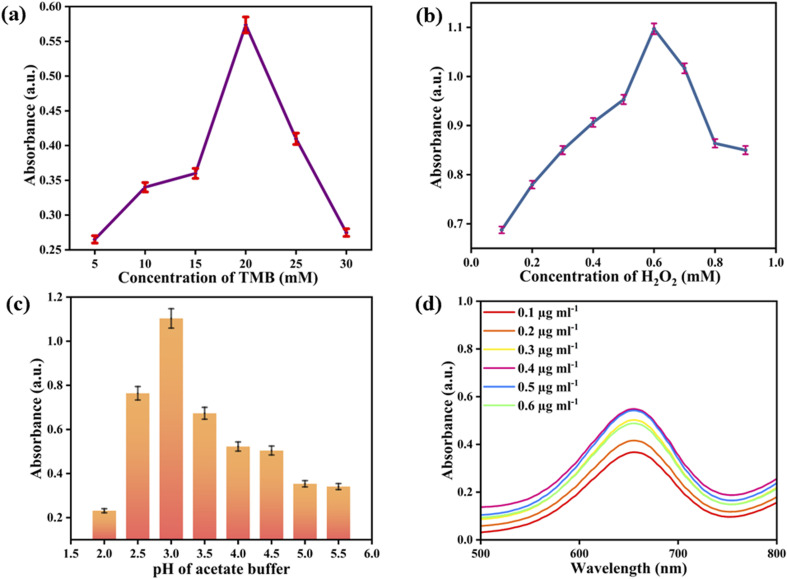
Optimization of key experimental parameters for the PUC-15@MCHS-based sensing system: effect of TMB concentration (a); effect of H_2_O_2_ concentration (b); effect of pH using acetate buffer (c); and effect of PUC-15@MCHS composite concentration (d).

To get the best results from the system, essential reaction parameters were systematically optimized. Adjustment of concentrations indicated that 20 mM TMB and 0.6 mM·H_2_O_2_ yielded the highest response, as shown in Fig. 10a and b. The effect of solution pH was also investigated using an acetate buffer (25 mM), in which pH 3 was found to be the optimum condition for maximum catalytic activity (Fig. 10c).

Finally, the nanozyme concentration was optimized, and a dosage of 0.4 µg mL^−1^ was determined to give the most pronounced results, as shown in Fig. 10d. These refined conditions were applied throughout the investigations for best outcomes.

### 3.8 Selectivity evaluation of the nanozyme system toward Hg^2+^ ions

**Fig. 11:**
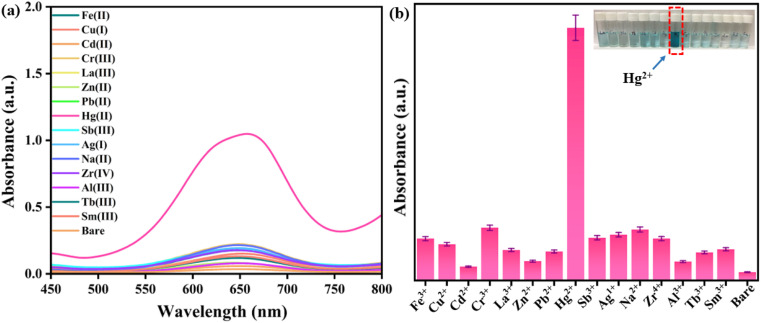
Absorption spectrum response of the PUC-15@MCHS-based sensing platform during TMB oxidation; evaluation of selectivity in the presence of various cations, showing a distinct response toward Hg^2+^ (a); bar graph of relative absorbance at 652 nm for each ion, indicative of oxidized TMB (b) (inset: visible colorimetric change observed only for Hg^2+^).

To evaluate the selectivity of PUC-15@MCHS towards diverse metal ions, experiments were conducted using various metal ions (Fe^2+^, Cu^2+^, Cd^2+^, Cr^3+^, La^3+^, Zn^2+^, Pb^2+^, Sb^3+^, Ag^+^, Na^+^, Zr^4+^, Al^3+^, Tb^3+^, Sm^3+^), with absorbance monitored at 652 nm. Among all the metal ions, Hg^2+^ induces a pronounced enhancement in the characteristic plasmonic band, whereas all other metal ions produce negligible changes in absorbance, as shown in Fig. 11a. The reaction system containing Hg^2+^ ion shows a dark greenish-blue colour compared to other ions shown in the inset of 11b. Bar graph comparison of the absorbance intensity (11b) further highlights this preference, with Hg^2+^ signals exceeding those of other ions by more than an order of magnitude, indicative of outstanding selectivity. It is observed that pristine MOF also displayed Hg^2+^ selectivity, but colour alteration is much more subtle, as shown in Fig. (S17).

### 3.9 Assessment of nanozyme-based sensing performance for Hg^2+^ detection

**Fig. 12:**
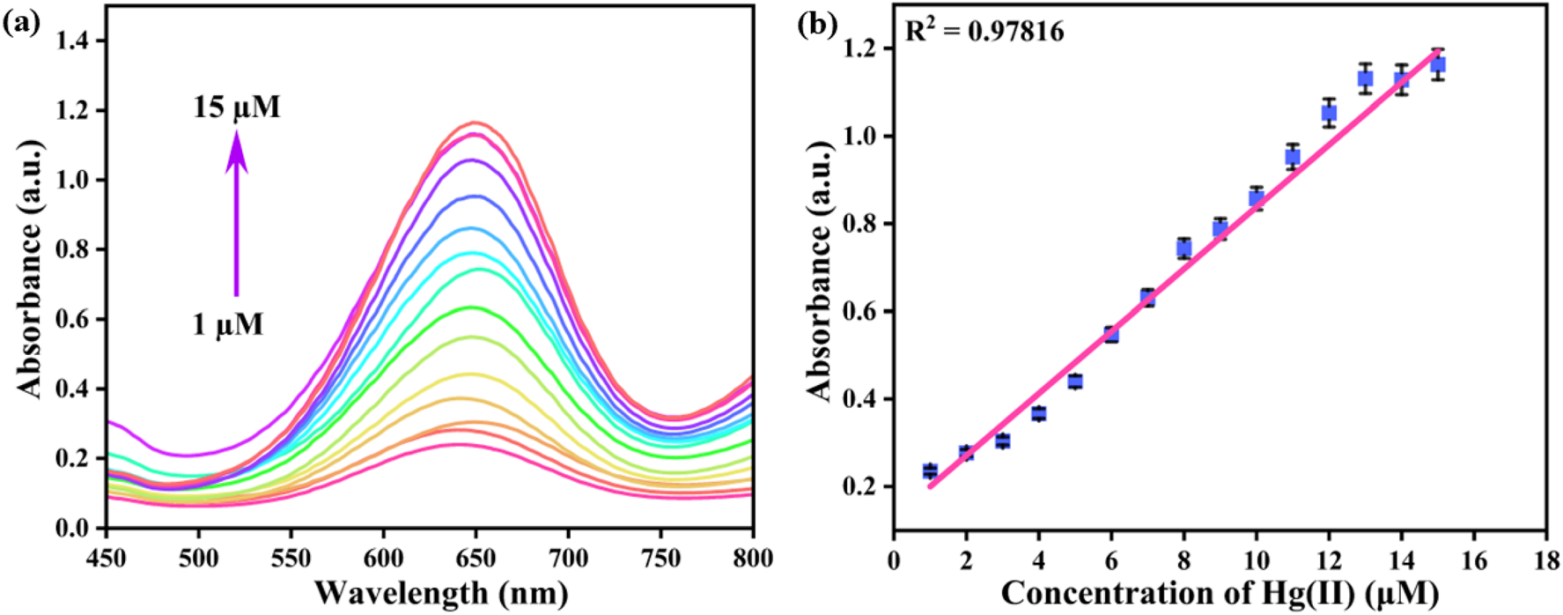
Absorption spectra showing catalytic oxidation of TMB with increasing Hg(ii) concentrations (1–15 µM), marked by enhanced absorbance at 652 nm; (a) linear calibration curve with *R*^2^ = 0.978 and slope = 0.07096 (b), with the limit of detection (3*σ*/*m*) estimated as 0.59 µM, confirming the detection of Hg^2+^ ions.

The analytical performance of nanozyme sensor PUC-15@MCHS was evaluated for Hg^2+^ sensing under optimized conditions. Upon gradual addition of Hg^2+^, a systematic increment in the absorbance pattern was observed at a wavelength of 652 nm. The reaction was accompanied by the colour change because the oxidation reaction of TMB generates a blue colour product, which was proportional to the concentration of Hg^2+^. A calibration curve was constructed using colorimetric absorbance across Hg^2+^ concentrations from 1 µM to 15 µM. A linear correlation was obtained over the investigated range, with a slope of 0.07096 and a correlation coefficient (*R*^2^) of 0.978, demonstrating consistent sensing behaviour as depicted in Fig. 12. The limit of detection was calculated using the relation LOD = 3*σ*/*m*, where *σ* is the standard deviation of the intercept (0.014), resulting in a detection limit of 0.59 µM, a comparison data of the present study with previously reported nanozyme-based systems for Hg^2+^ sensing is provided in (Table S5). The satisfactory sensitivity of the system with the feature of on-spot visual detection makes it suitable and convenient for monitoring mercury ions in environmental samples. For practical evaluation, Table S6 compares the present system with representative reported Hg^2+^ sensors in terms of sensing medium, pH operating window, response time, and selectivity.

### 3.10 Steady-state enzyme kinetics and catalytic evaluation of PUC-15@MCHS nanozyme

**Fig. 13:**
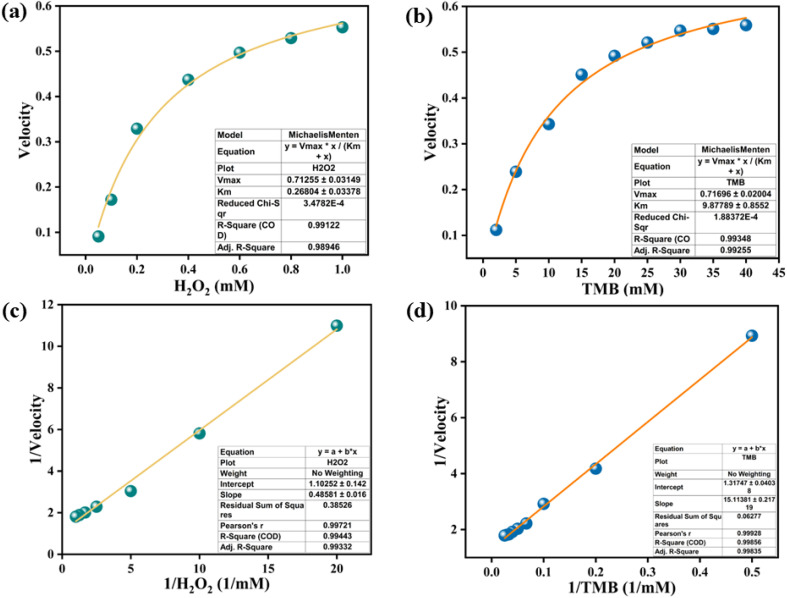
Steady-state kinetics of the PUC-15@MCHS nanozyme with TMB and H_2_O_2_ substrates: Michaelis–Menten plots illustrating catalytic oxidation rates for H_2_O_2_ and (a) for TMB; (b) Lineweaver–Burk plots confirming classical Michaelis–Menten kinetics for H_2_O_2_ and (c) for TMB (d).

Steady-state kinetic analysis was conducted to elucidate the catalytic mechanism of the PUC-15@MCHS nanozyme. Initial reaction velocities (*V*_0_) were calculated using the relation (*Δ* Absorbance)/time, monitored at 652 nm for oxidized TMB formation. The kinetic data were measured by fixing the concentration of H_2_O_2_ and varying TMB concentrations or *vice versa* with PUC-15@MCHS. The steady-state kinetics of TMB oxidation by H_2_O_2_ catalyzed with PUC-15@MCHS are shown in Fig. 13. The kinetic data show a typical Michaelis–Menten curve, as shown in Fig. 13a and b. Then the corresponding double reciprocal plots (Lineweaver–Burk plots) were plotted as shown in Fig. 13(C and D). The equations used for Michaelis–Menten and Lineweaver–Burk plots were eqn (1) and (2), respectively, where *V*_0_ and *V*_max_ represent the initial and maximum reaction rate. It was observed that the reaction rate of TMB oxidation gradually increased with increasing H_2_O_2_ concentration while keeping the TMB concentration constant. However, after reaching a certain H_2_O_2_ concentration, the rate of increase slowed down and eventually levelled off. Similarly, when the concentration of H_2_O_2_ was kept constant, the peroxidase-like reaction rate of PUC-15@MCHS increased with increasing TMB concentration (Fig. 6B). Nevertheless, once the TMB concentration exceeded a certain limit, the reaction rate began to slow down. The kinetic parameters, *K*_m_ and *V*_max_, were determined from the intercept and slope of the linear plot. The *K*_m_ values indicate the affinity of an enzyme towards the substrate, which was found to be lower for H_2_O_2_ than TMB, underscoring the substrate preference of the nanozyme.

The hyperbolic relationship between (*V*_0_) and [*S*] confirms the catalytic nature of the nanozyme. Nonlinear regression analysis provided *K*_m_ and *V*_max_ values of 9.88 mM and 0.717 mM min^−1^ for TMB, while corresponding values of 0.27 mM and 0.713 mM min^−1^ were obtained for H_2_O_2_. Furthermore, the Lineweaver–Burk reciprocal plots (Fig. (13c and d) demonstrated excellent linearity between 1/*V*_0_ and 1/[*S*], with correlation coefficients *R*^2^ exceeding 0.99, reinforcing the applicability of the Michaelis–Menten kinetic framework to this catalytic system.1
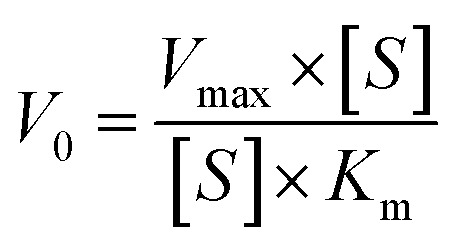
2
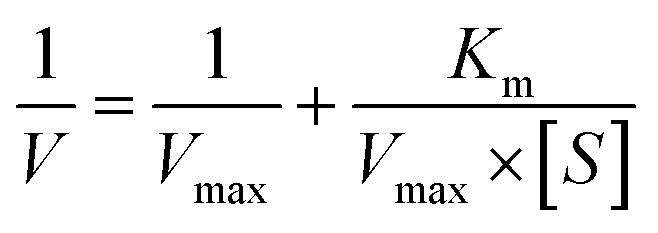


Collectively, these results are consistent with established enzyme kinetics methodologies commonly employed in nanozyme research^67,68^ and confirm that the PUC-15@MCHS nanozyme exhibits highly efficient peroxidase-mimic activity and superior substrate binding characteristics. To evaluate the intrinsic catalytic performance of the framework, the peroxidase-like activity of pristine PUC-15 was also investigated under identical experimental conditions. The corresponding results are provided in Fig. S18. The *K*_m_ values were further compared with those of natural HRP and previously reported nanozymes to place the substrate affinity of the present system in a broader catalytic context (Table S7).

## 4 Assessment of PUC-15@MCHS efficacy in different water samples for UV-visible colorimetric detection of Hg(ii)

The applicability of the colorimetric approach for detecting Hg(ii) was investigated on water samples from various sources: distilled water (DW), tap water from Chandigarh (CTW), Gagghar river water (RW), groundwater (GW), and Industrial pharmaceutical water (IPW). Before testing, samples were passed through a Whatman Grade 42 filter paper to remove suspended solids and were kept at ambient temperature. For this purpose, the samples were spiked with different concentrations of Hg(ii) (1, 5, and 10 µM), representing low, medium, and high levels relative to the method's limit of detection (0.59 µM). The unspiked samples were used as blanks. The experiment was performed twice in triplicate, and the results are reported as mean values with relative standard deviation (RSD). From titration experiments with UV-visible spectroscopic detection, the found Hg^2+^ concentrations were calculated, and a comparison between the experimental results and spiked ones was enumerated in Table S8. The recovery results for Hg(ii)-spiked samples are found to be reliable and consistent across different water matrices. It is observed that the blank samples showed stable recoveries with minimal deviation, confirming the reliability of the baseline measurements. Collectively, these results highlight the practical feasibility of the PUC-15@MCHS nanozyme composite in sensing and monitoring of Hg^2+^.

## 5 Mechanism for mercury ion sensing *via* nanozyme-mimicking activity

**Fig. 14:**
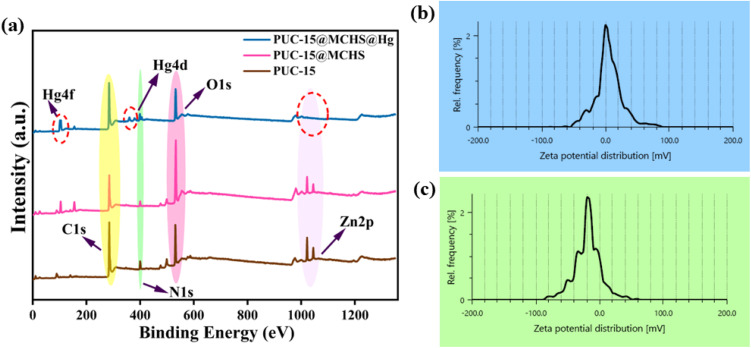
XPS and zeta potential analysis of PUC-15 and PUC-15@MCHS: XPS survey spectra before and after Hg^2+^ sensing, illustrating surface composition changes (a); zeta potential of PUC-15 (b); zeta potential of PUC-15@MCHS (c).

To get insight into the mechanism of Hg^2+^ sensing, various characterization techniques have been employed. The nanozyme PUC-15@MCHS behaves as a turn on colorimetric sensor, exhibiting enhanced enzymatic activity in the presence of Hg^2+^. The nanozyme exhibits peroxidase-like activity by catalysing H_2_O_2_-facilitated oxidation of TMB. The mechanism of this peroxidase-like activity is similar to Fenton-like chemistry, where H_2_O_2_ adsorbs onto the metal sites of the material to generate hydroxyl radicals, followed by oxidation of TMB to blue-coloured oxTMB. To identify the dominant reactive oxygen species involved in the Hg^2+^ -enhanced catalytic process, radical trapping experiments were performed using isopropanol and benzoquinone as selective scavengers for ˙OH radical and O_2_˙^−^ radical, respectively. As shown in Fig. S19, the addition of isopropanol markedly suppressed the TMB oxidation signal, whereas benzoquinone caused only partial inhibition, indicating that OH radicals are the major reactive species responsible for the enhanced catalytic response, while O_2_˙^−^ radicals play a comparatively minor role. Upon Hg^2+^ addition, catalytic efficiency increases markedly, which may be attributed to strong surface interaction and possible surface modulation of the nanozyme. Firstly, Hg^2+^ interacts with electron-rich sites of the nanozyme surface, leading to the formation of surface-associated Hg species, possibly including partially reduced Hg-containing clusters. XPS study of the nanozyme before and after reaction with Hg^2+^ was conducted to investigate the interaction. The Zn 2p peaks at 1021.8 and 1044.9 eV, originating from Zn^2+^ coordinated to carboxylate groups, shift upon Hg^2+^ exposure, as shown in Fig. 14a. Simultaneously, new peaks corresponding to Hg 4f_7/2_ (100.0 eV) and Hg 4f_5/2_ (104.1 eV) appear in the spectrum, indicating the presence of Hg-containing species on the material surface. Further insights from high-resolution N 1s and O 1s spectra show shifts of 0.3–0.5 eV toward higher binding energies after Hg^2+^ addition, suggesting coordination of Hg^2+^ to pyridyl nitrogen and carboxylate oxygen sites. To further determine whether the Hg-derived species were weakly adsorbed or remained associated with the composite after sensing, washing-control experiments were performed. As shown in Fig. S20, the Hg-induced absorbance feature was retained even after thorough washing of the Hg-treated sample, indicating that the Hg-derived species remained immobilized on the surface rather than existing as free ions in solution. Moreover, the washed Hg-treated sample still exhibited enhanced catalytic activity, further supporting that these surface-associated Hg-derived species persist after washing and continue to promote the catalytic process. In addition, post-sensing HRTEM analysis of the washed Hg-treated PUC-15@MCHS sample revealed nanoscale surface clusters that were absent in the pristine material, supporting the formation of Hg-derived surface deposits under sensing conditions (Fig. S21). SEM analysis indicates that the composite retains a porous morphology, which can facilitate contact between Hg^2+^ ions, H_2_O_2_, TMB, and the catalytic surface. In addition, zeta potential measurements reveal a negative surface charge (−18.6 mV) on the nanozyme, which favors electrostatic attraction of positively charged ions (Fig. 14c and d). The Hg-derived species formed on the surface are proposed to modulate the local catalytic environment and thereby enhance reactive oxygen species generation, leading to more efficient oxidation of TMB. This interpretation is consistent with the steady-state kinetic analysis, which shows a significantly lower *K*_m_ value toward H_2_O_2_ after Hg^2+^ treatment. As a result, the presence of Hg^2+^ produces a rapid and distinct increase in the blue coloration of oxidized TMB, giving rise to the turn-on colorimetric sensing response of the nanozyme. Collectively, these results suggest that the enhanced peroxidase-like activity of the nanozyme underlies its rapid and sensitive colorimetric sensing performance.

## 6 Conclusion

In this study, a colorimetric sensor based on the peroxidase-mimicking activity was developed for the detection of Hg^2+^. A novel zinc-based metal–organic framework (PUC-15) was synthesized *via* solvothermal methods and composited with mesoporous carbon hollow spheres (MCHS) to enhance performance. The composite (PUC-15@MCHS) formed with an average pore size of about 4.64 nm and a surface area of 84.18 m^2^ g^−1^. In the presence of H_2_O_2_, PUC-15@MCHS efficiently catalyzed TMB oxidation, producing a rapid color change. This property enabled a reliable colorimetric assay for Hg^2+^. Validation in real samples confirmed the sensor's practicality. These findings highlight the potential of PUC-15@MCHS peroxidase-like nanozyme activity in catalysis, biosensing, and environmental monitoring.

## Conflicts of interest

The authors state that they have no competing financial interests or personal relationships that could have affected the outcomes of this work.

## Supplementary Material

RA-OLF-D6RA02355J-s001

RA-OLF-D6RA02355J-s002

## Data Availability

CCDC 2497354 (PUC-15) contains the supplementary crystallographic data for this paper.^69^ All the details are available within the published article and supplementary information (SI) file. Supplementary information is available. See DOI: https://doi.org/10.1039/d6ra02355j.
