# Supplementary material for: Zinc-MOF-carbon hollow sphere hybrid nanozyme for highly selective colorimetric detection of mercury ions
Source: RSC Adv. 2026 Aug 6;16(39):45092–105. doi: 10.1039/d6ra02355j (PMC13446140; doi:10.1039/d6ra02355j)
Supplement: Supplementary file 1 [file RA-016-D6RA02355J-s001.pdf]

Supplementary Information (SI) for RSC Advances.
This journal is © The Royal Society of Chemistry 2026

Zinc-MOF-Carbon Hollow Sphere Hybrid Nanozyme for Highly Selective Colorimetric Detection of Mercury Ions

Garima,[a] Kushal Arya,[a,b] Indu Sharma,[a] Ennio Zangrando,[c] Vinod Kumar[d], Rakesh Kumar Bhardwaj[d] and Ramesh Kataria[a,]*

[a] Department of Chemistry and Centre of Advanced Studies in Chemistry, Panjab University, Chandigarh-160014, India

[b] University Center for Research and Development, Chandigarh University, Mohali, Punjab-140301, India

[c] Department of Chemical and Pharmaceutical Sciences, University of Trieste, Trieste 34127, Italy

[d] Department of Chemistry, Central University of Haryana, Mahendragarh-123029, India

**Email: rkataria@pu.ac.in*

Fig. No.	Captions
**S1**	EDS Comparison spectra.
**S2**	TGA profiles of pristine MOF, MCHS, and MOF@MCHS composites.
**S3**	The histogram corresponds to the size distribution derived from TEM images for PUC-15@MCHS.
**S4**	Elemental Mapping of PUC-15 and PUC-15@MCHS.
**S5**	Carbon mapping of PUC-15 and PUC-15@MCHS.
**S6**	Nitrogen mapping of PUC-15 and PUC-15@MCHS.
**S7**	Oxygen mapping of PUC-15 and PUC-15@MCHS.
**S8**	Zinc mapping of PUC-15 and PUC-15@MCHS.
**S9**	XPS spectra of C1s and O1s in composite (PUC-15@MCHS).
**S10**	XPS spectra of N1s and Zn2p in composite (PUC-15@MCHS).
**S11**	XPS spectra of N1s and C1s in composite (PUC-15@MCHS) after Hg detection.
**S12**	XPS spectra of O1s and Zn2p in composite (PUC-15@MCHS) after Hg detection.
**S13**	XPS spectra of Hg4f and Hg4d.
**S14**	(a) UV-vis of TMB oxidation (652 nm). (b) Time-dependent H_2O_2-dependent nanozyme activity.
**S15**	Absorbance spectra of MCHS, MOF, their physical mixture (PUC-15+MCHS), and the composite PUC-15@MCHS, demonstrating the change in optical behavior after composite formation.
**S16**	Comparative catalytic activity of MOF, MCHS, and PUC-15@MCHS composites with varying compositions.
**S17**	Bar graph illustrating the comparative catalytic activity of different materials: (1) Bare system (without catalyst), (2) pristine MOF (PUC-15), and (3) composite (PUC-15@MCHS).
**S18**	Steady-state kinetic analysis of pristine PUC-15 toward TMB oxidation in the presence of H_2O_2.
**S19**	UV-Vis absorption spectra of the TMB/H_2O_2 system in the presence of PUC-15@MCHS, Hg^{2+}, and radical scavengers (isopropanol and benzoquinone),

**S20**	Comparative absorption spectra of PUC-15@MCHS, PUC-15@MCHS + Hg^{2+} (unwashed), and Hg-treated PUC-15@MCHS (washed), indicating the effect of Hg^{2+} interaction on the optical behavior of the composite.
**S21**	HR-TEM images of the Hg-treated PUC-15@MCHS.

**Sr. No.**	**Tables**
**Table S1**	Selected Bond lengths (Å) of PUC-15.
**Table S2**	Selected Bond angles (°) of PUC-15.
**Table S3**	Elemental composition of MCHS, pristine PUC-15, and PUC-15@MCHS composites.
**Table S4**	XPS binding energy shift comparison of C 1s, N 1s, O 1s, Zn $2p_{1/2}$ and Zn $2p_{3/2}$ for the MOF and composite.
**Table S5**	Comparative analysis of PUC-15@MCHS with reported Hg^{2+} sensors.
**Table S6**	Comparison of Hg^{2+} sensors based on practical performance parameters.
**Table S7**	Literature comparison of K_m values for TMB and H_2O_2 for natural horseradish peroxidase and selected peroxidase-like nanozyme catalysts.
**Table S8**	Real Water Sample Analysis for Hg^{2+} Detection Using PUC-15@MCHS Nanozyme Composite. (DW = Distilled water; CTW = Tap water from Chandigarh, RW = Gagghar River water, GW = Groundwater, and IPW = Industrial pharmaceutical water).

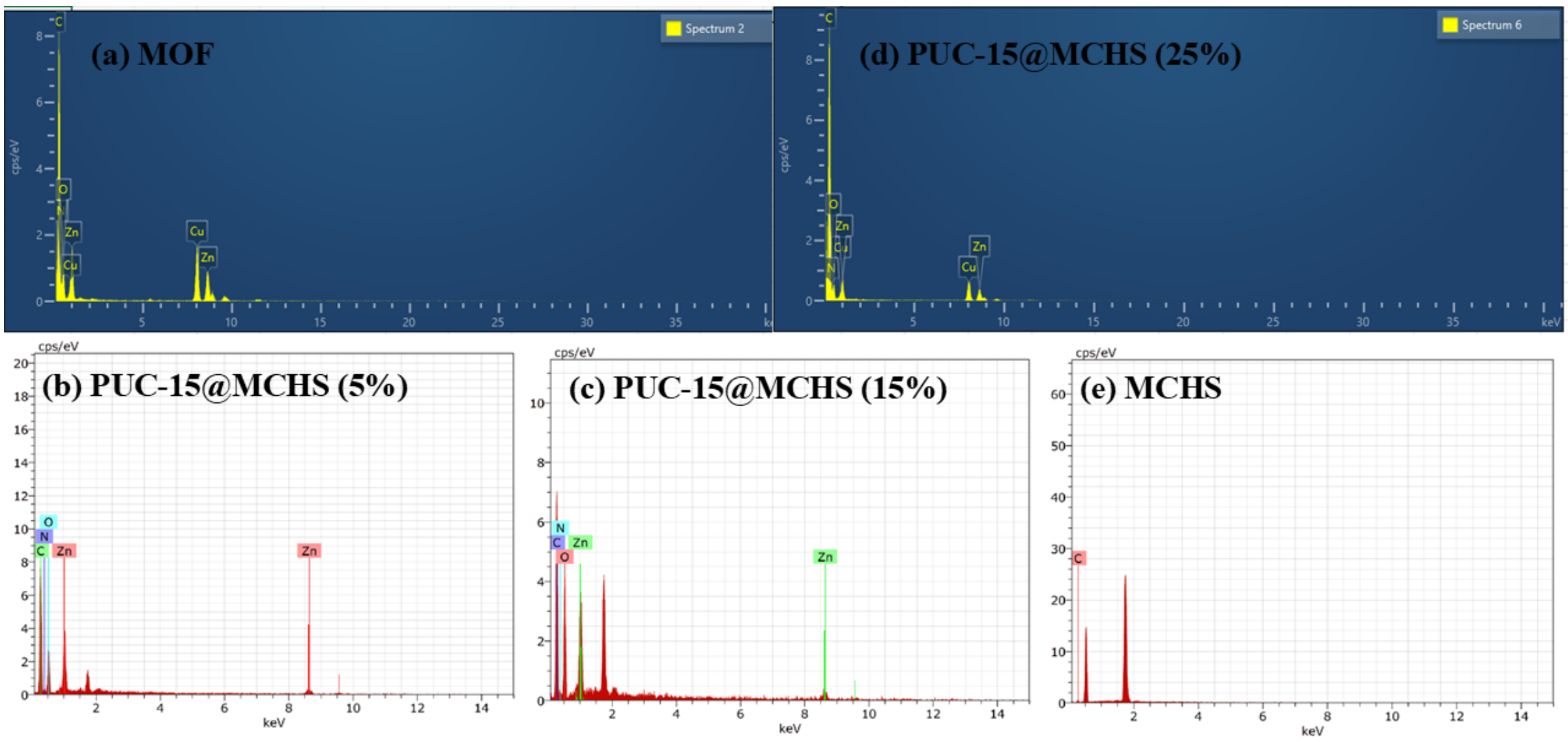

Figure S1. Comparative EDS spectra of pristine MOF, MCHS, and MOF@MCHS composites with different loadings, showing the elemental composition of the synthesized materials.

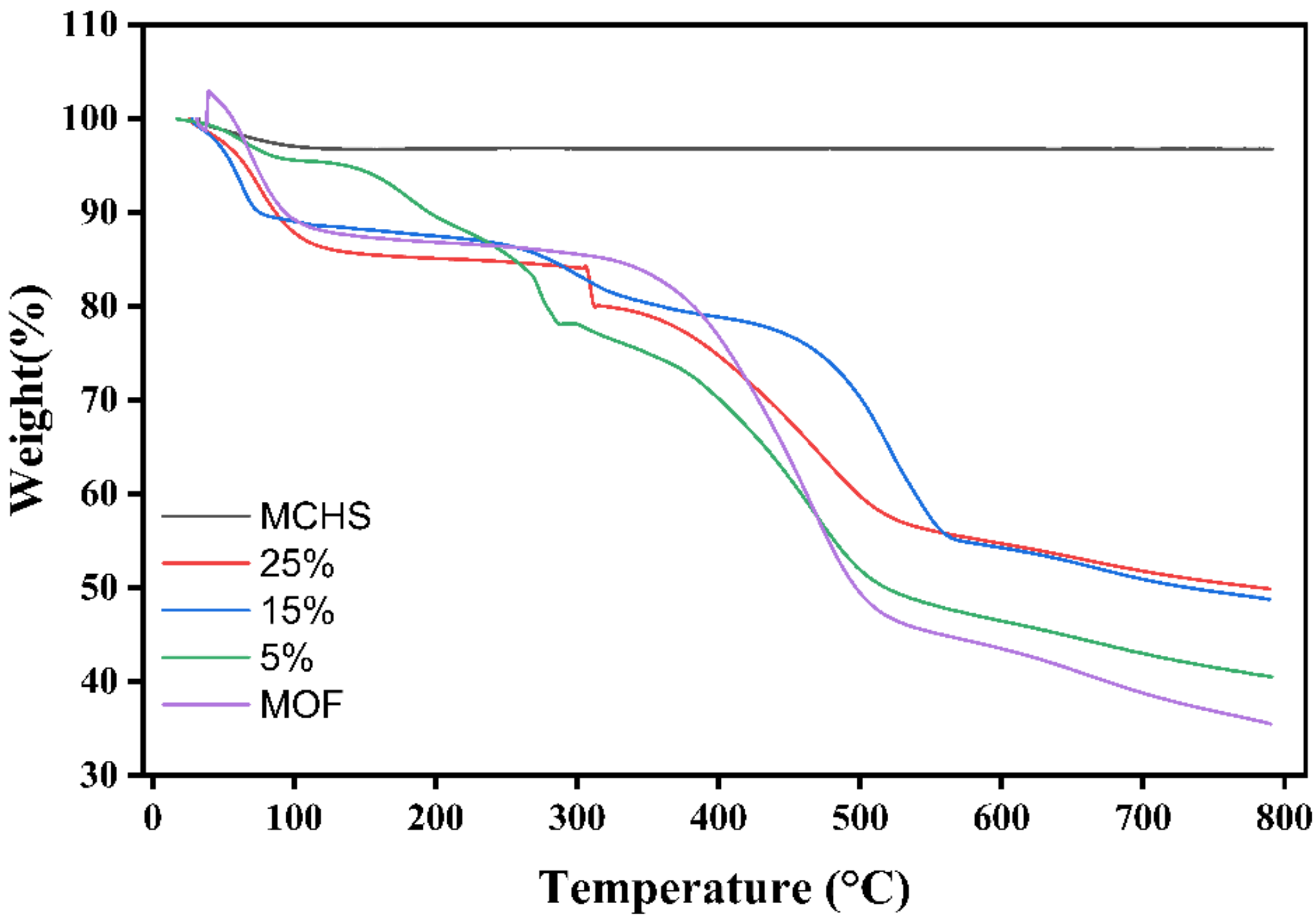

Figure S2. TGA profiles of pristine MOF, MCHS, and MOF@MCHS composites with different MCHS loadings (5%, 15%, and 25%), illustrating their thermal decomposition behavior and residual mass variation.

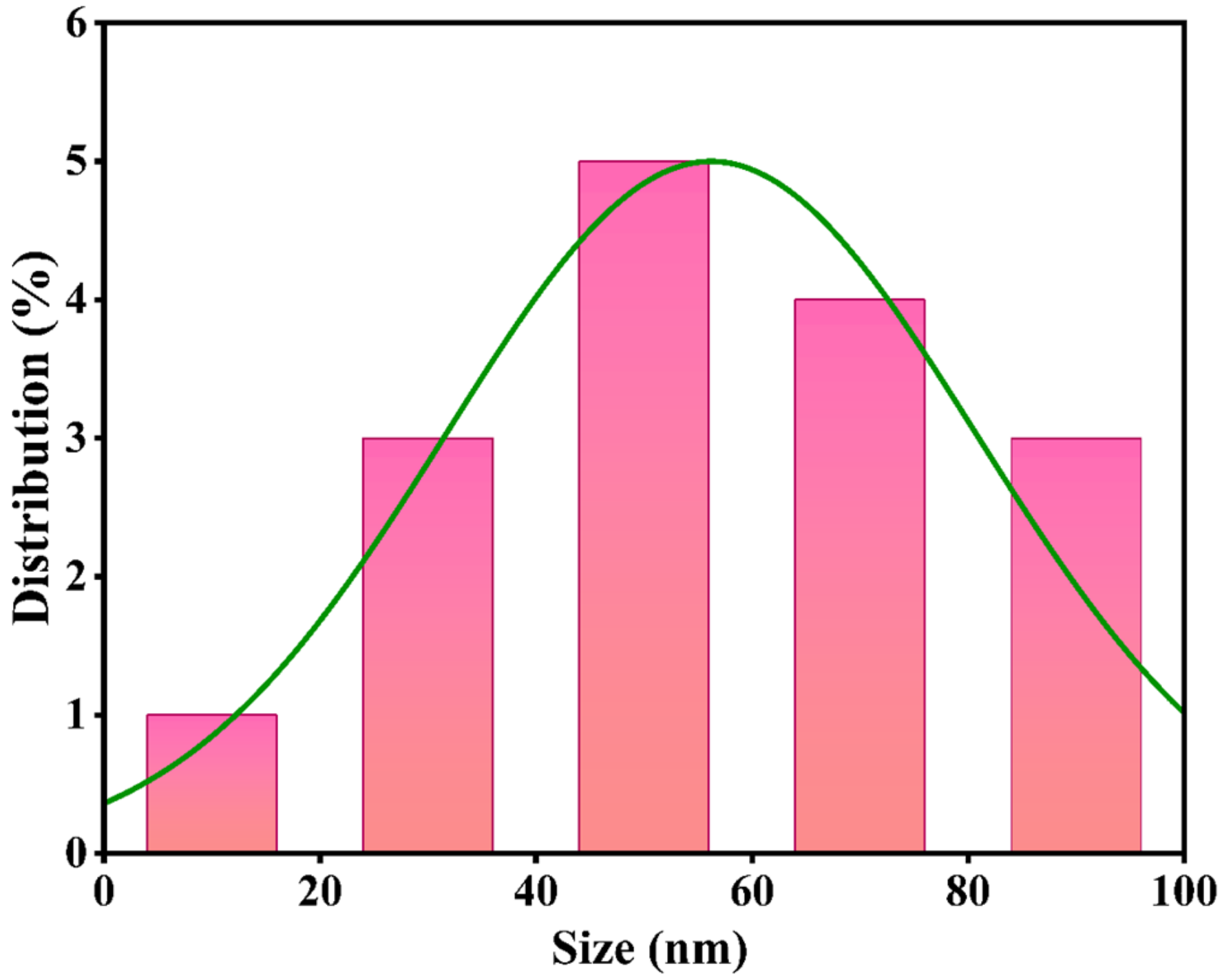

Figure S3. The histogram corresponds to the size distribution derived from TEM images for PUC-15@MCHS.

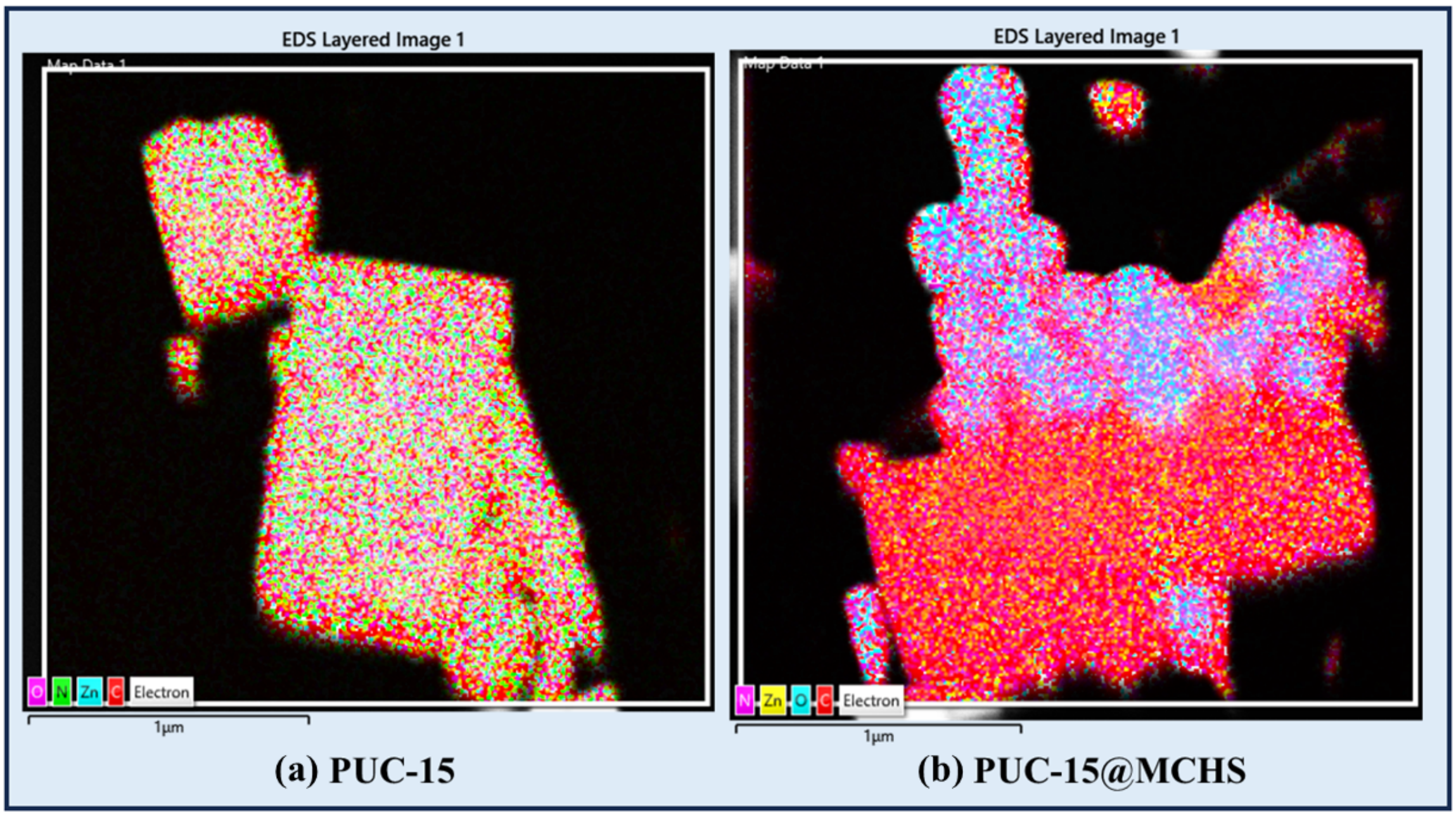

Figure S4. Elemental Mapping of the MOF (PUC-15) and Composite (PUC-15@MCHS).

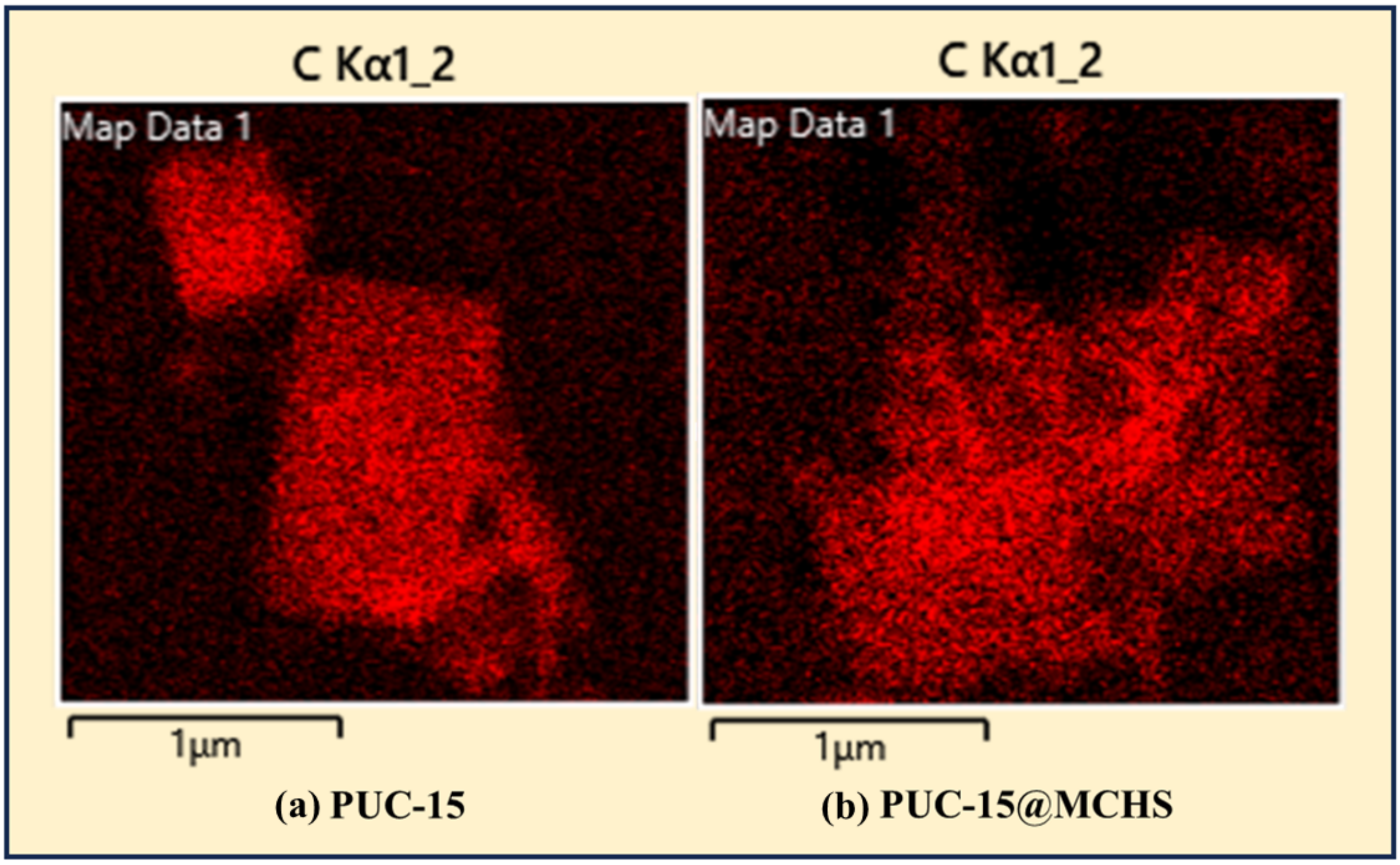

Figure S5. EDS elemental mapping of carbon (C) in the MOF (PUC-15) and its composite (PUC-15@MCHS).

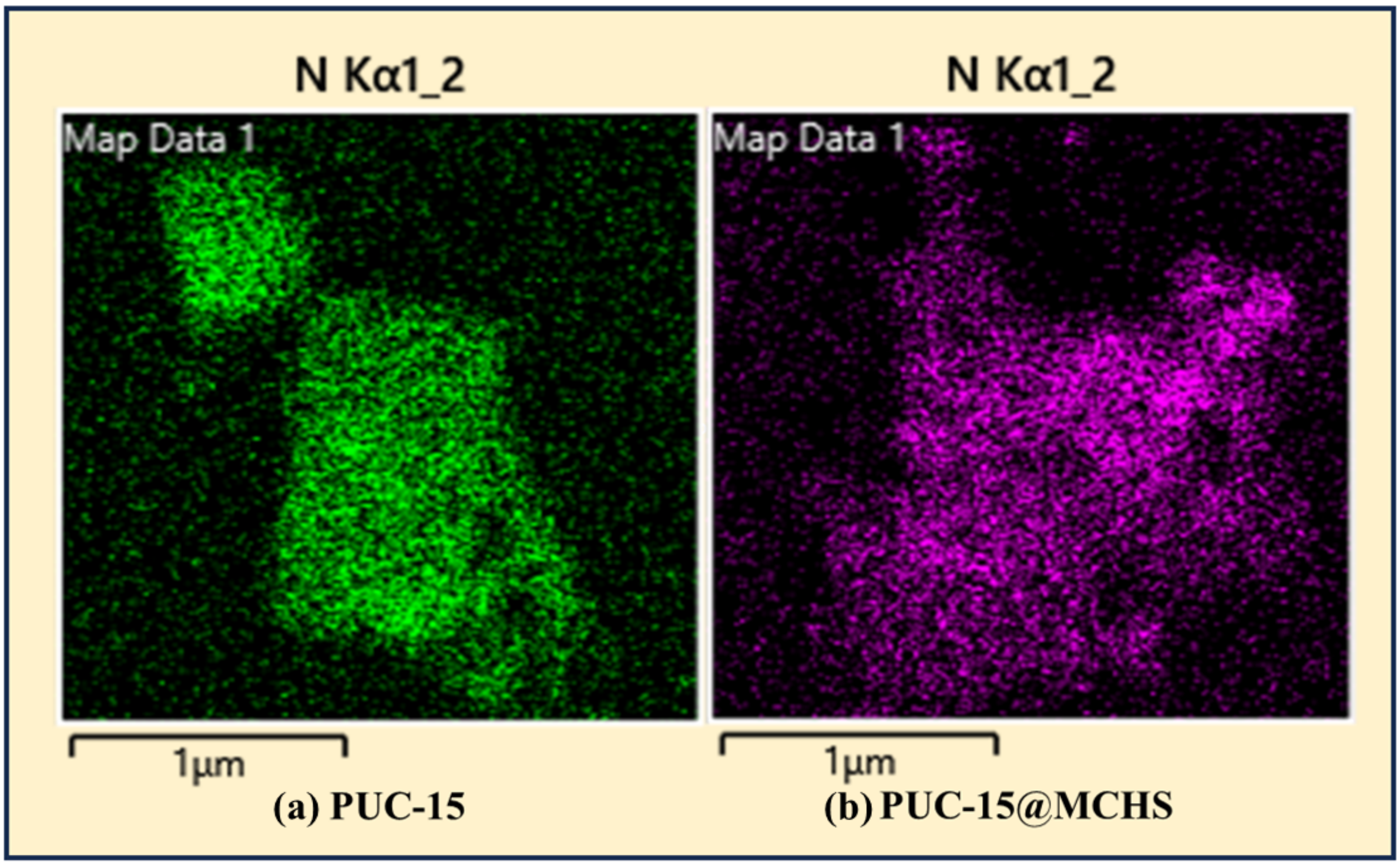

Figure S6. EDS elemental mapping of Nitrogen (N) in the MOF (PUC-15) and its composite (PUC-15@MCHS).

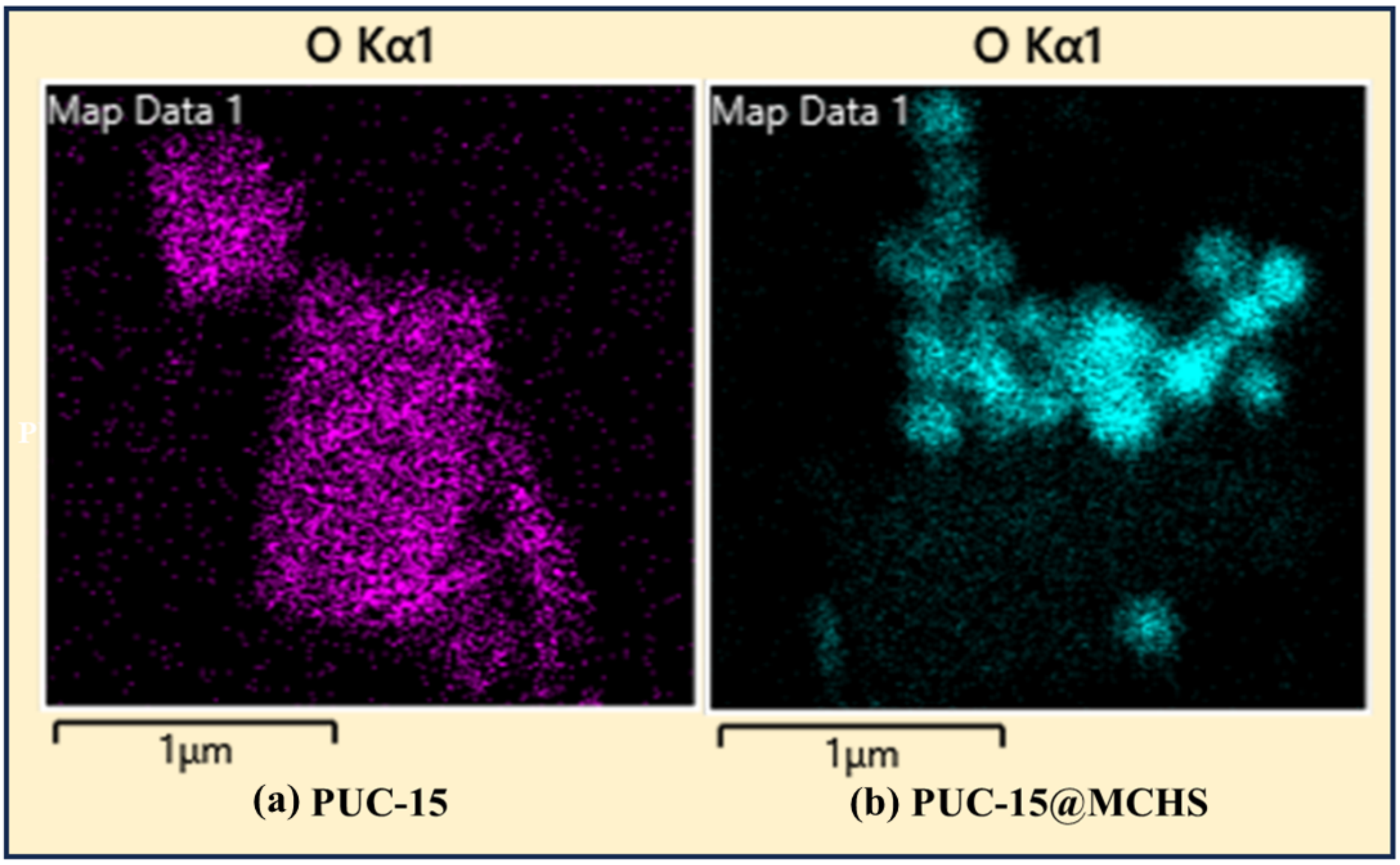

Figure S7. EDS elemental mapping of oxygen (O) in the MOF (PUC-15) and its composite (PUC-15@MCHS).

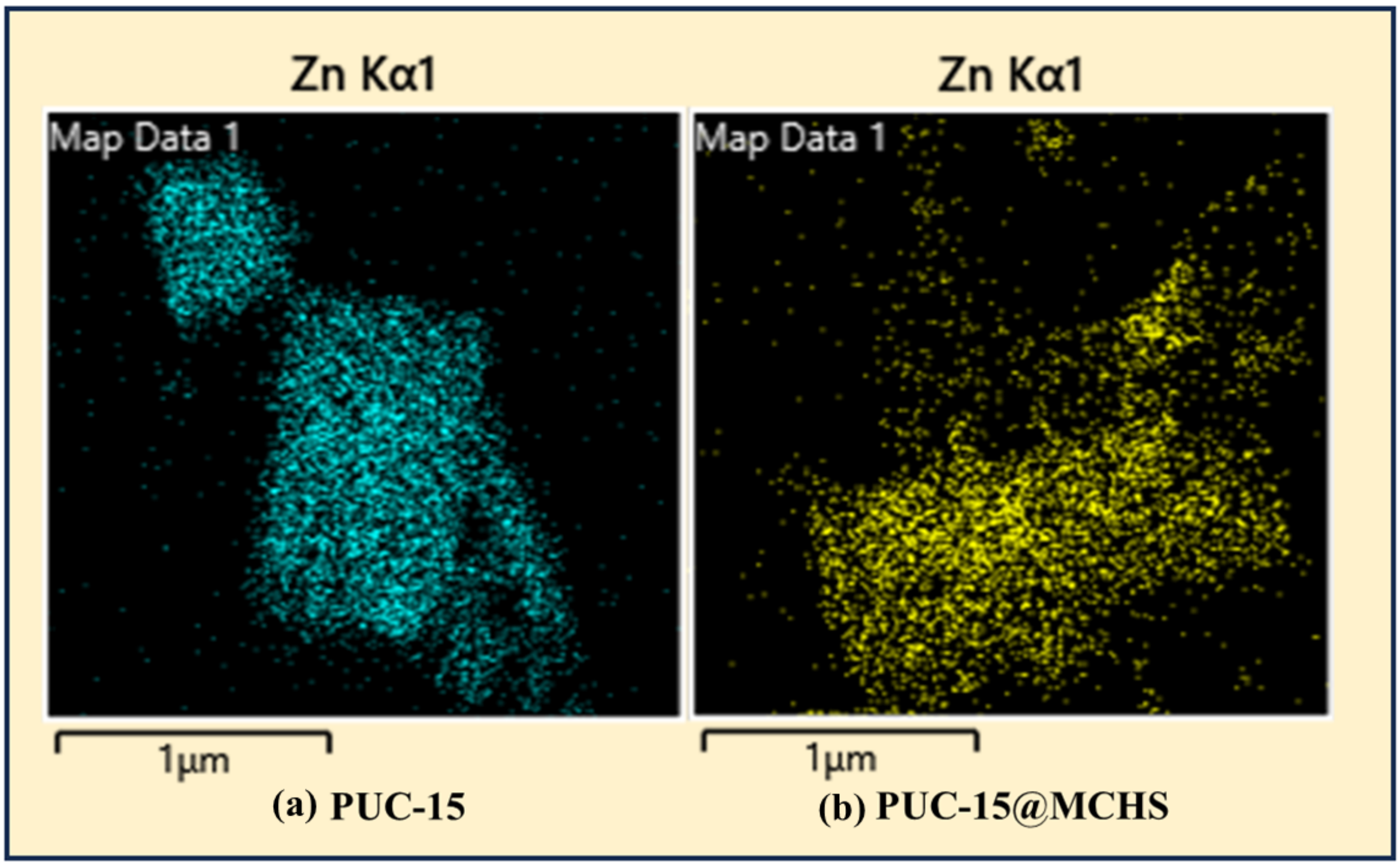

Figure S8. EDS elemental mapping of Zinc (Zn) in the MOF (PUC-15) and its composite (PUC-15@MCHS).

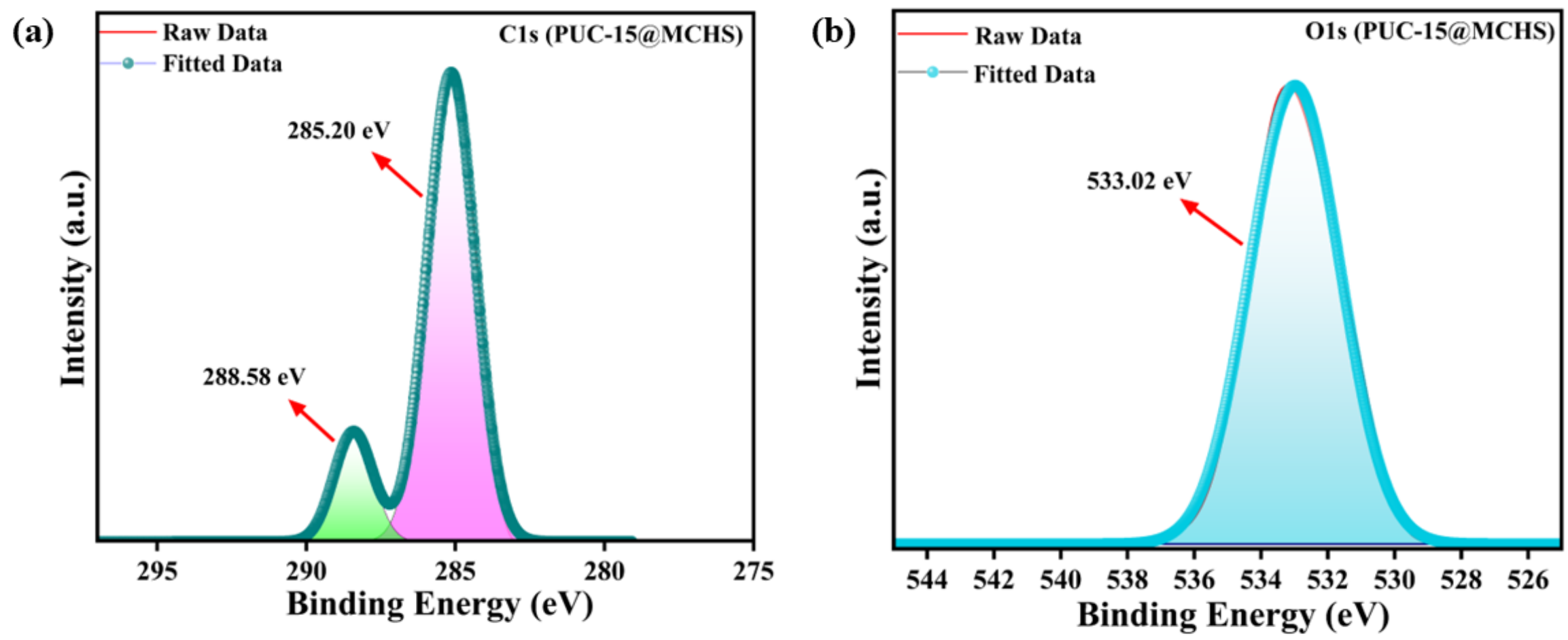

Figure S9. XPS spectra of the composite showing (a) C 1s and (b) O1s peaks, confirming the presence of nitrogen functionalities and Zn species.

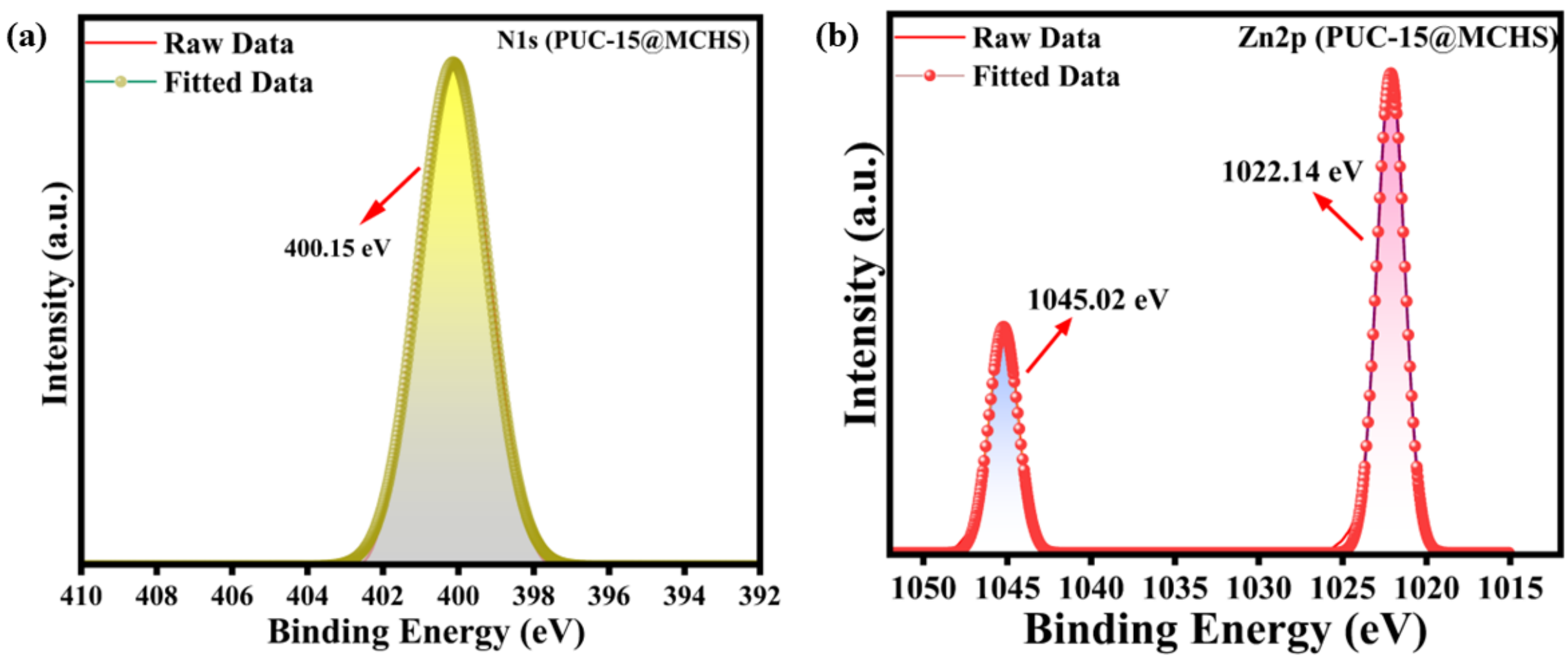

Figure S10. XPS spectra of the composite showing (a) C 1s and (b) Zn 2p peaks, confirming the presence of nitrogen functionalities and Zn species.

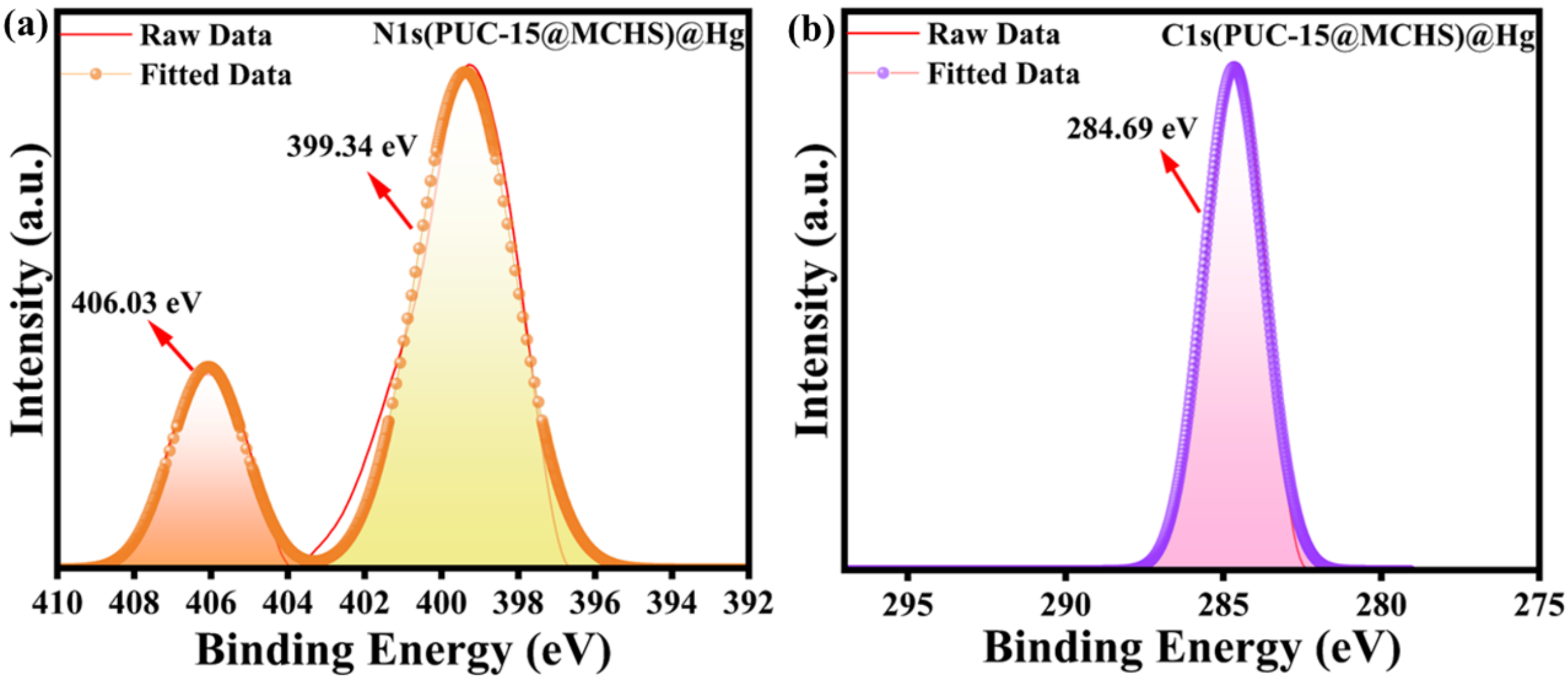

Figure S11. XPS spectra of nitrogen (N 1s) and carbon (C 1s) regions of the composite after Hg^{2+} detection.

.

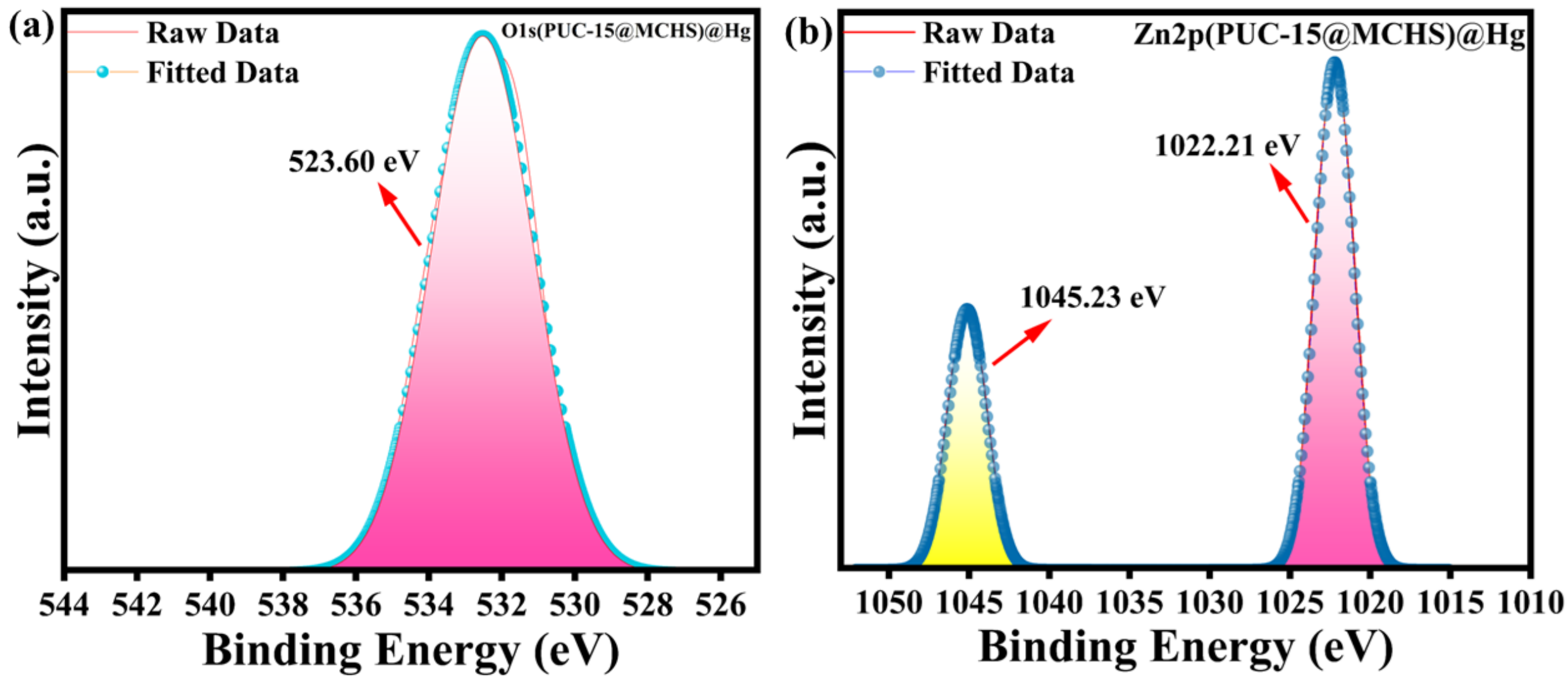

Figure S12. XPS spectra of oxygen (O 1s) and zinc (Zn 2p) regions of the composite after Hg^{2+} detection.

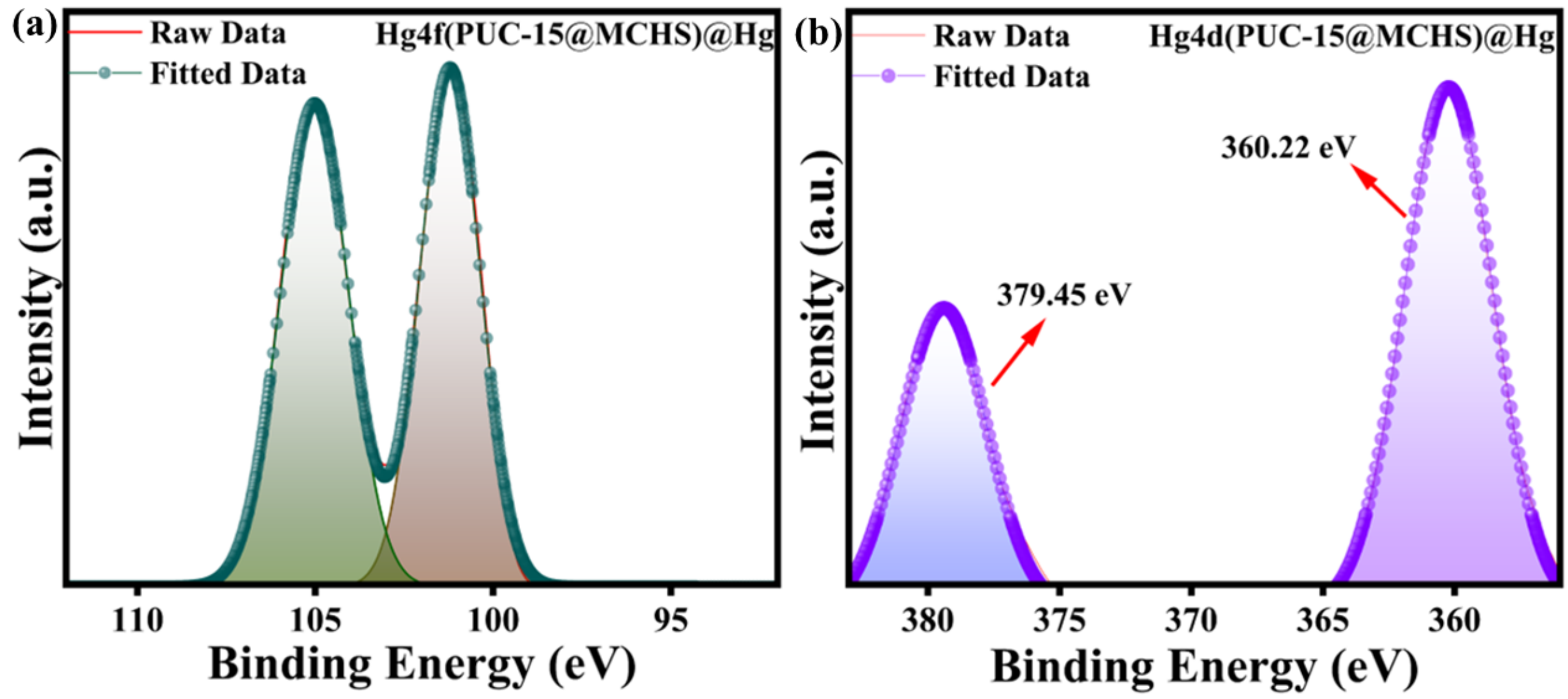

Figure S13. XPS spectra of Hg4f and Hg4d.

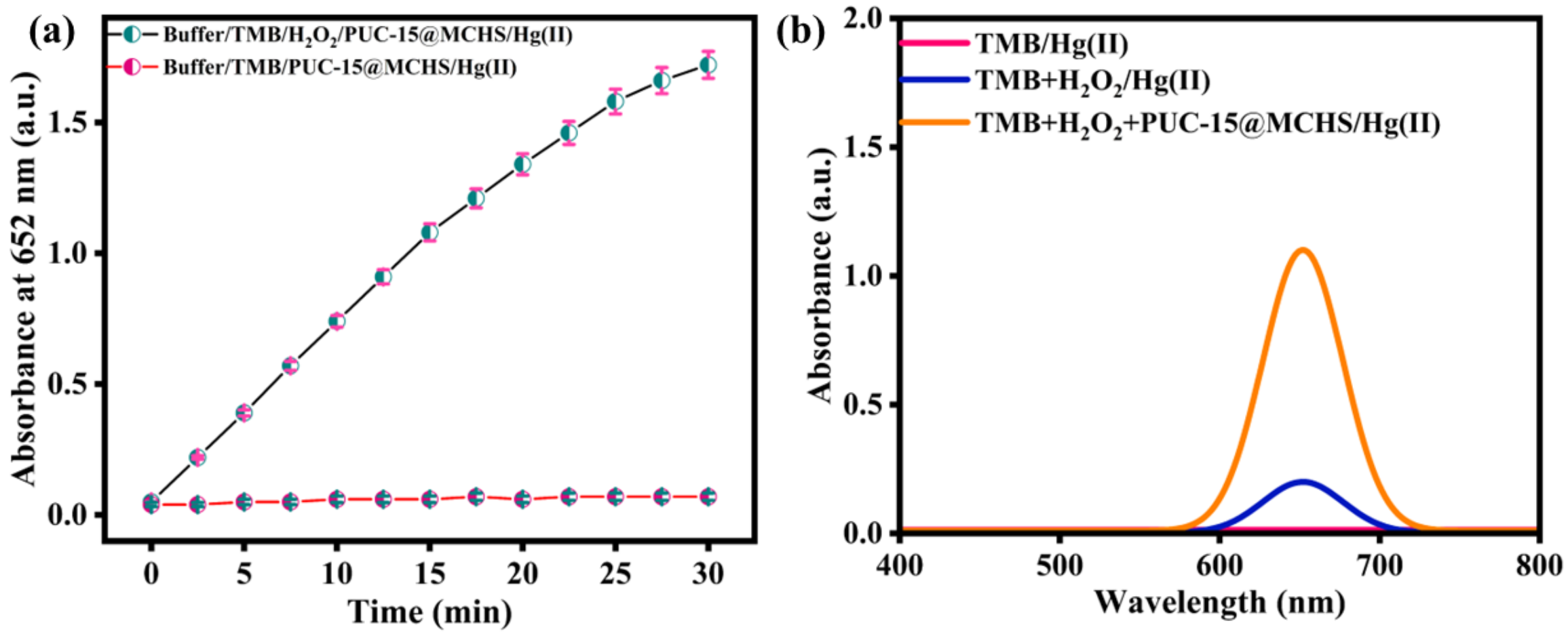

Figure S14. (a) Time-dependent study demonstrating H_2O_2-dependent peroxidase-like activity of the nanozyme. (b) UV-vis spectra of TMB oxidation catalyzed by the composite showing a 652 nm peak.

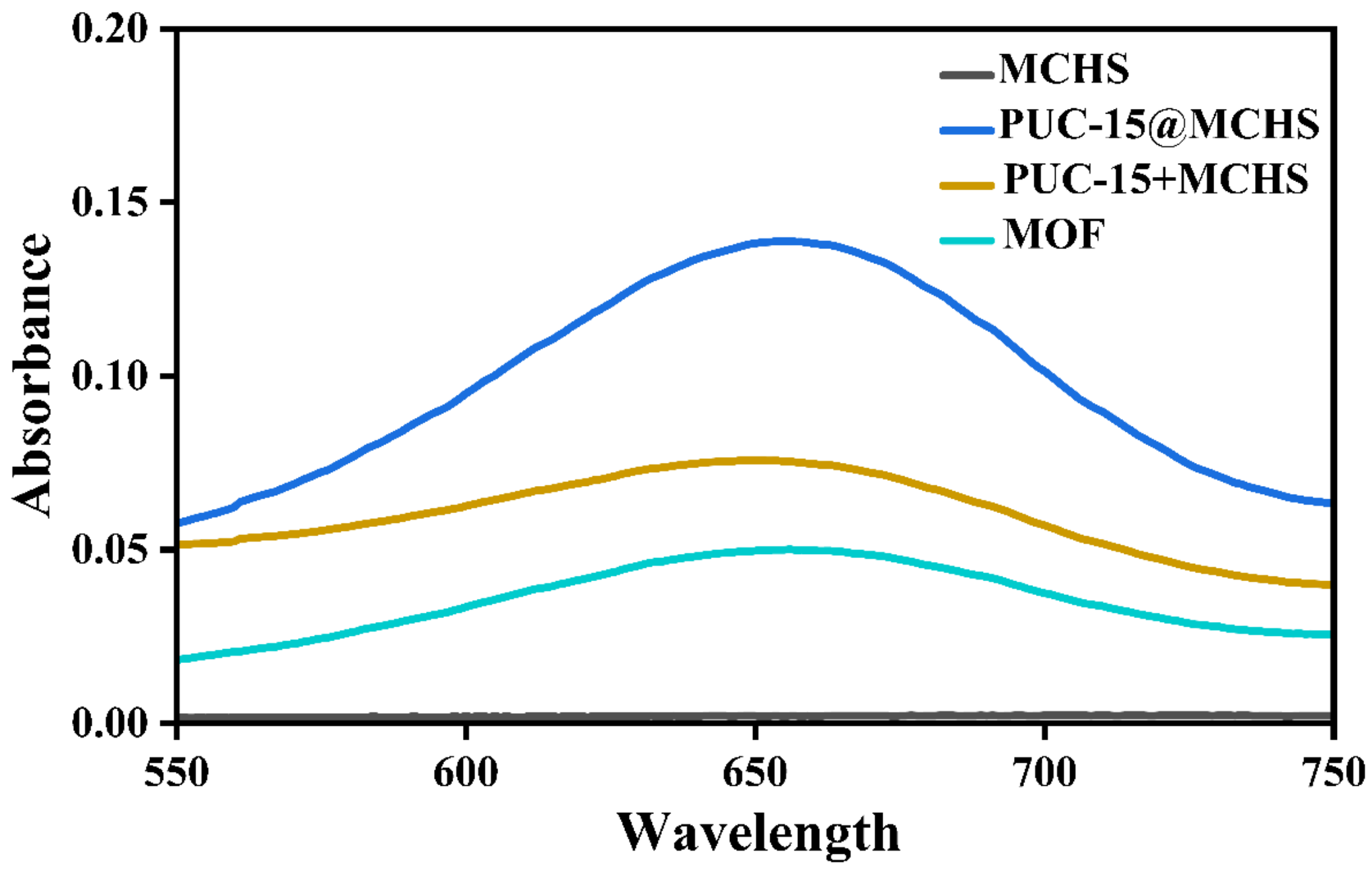

Figure S15. Absorbance spectra of MCHS, MOF, their physical mixture (PUC-15+MCHS), and the composite PUC-15@MCHS, demonstrating the change in optical behavior after composite formation.

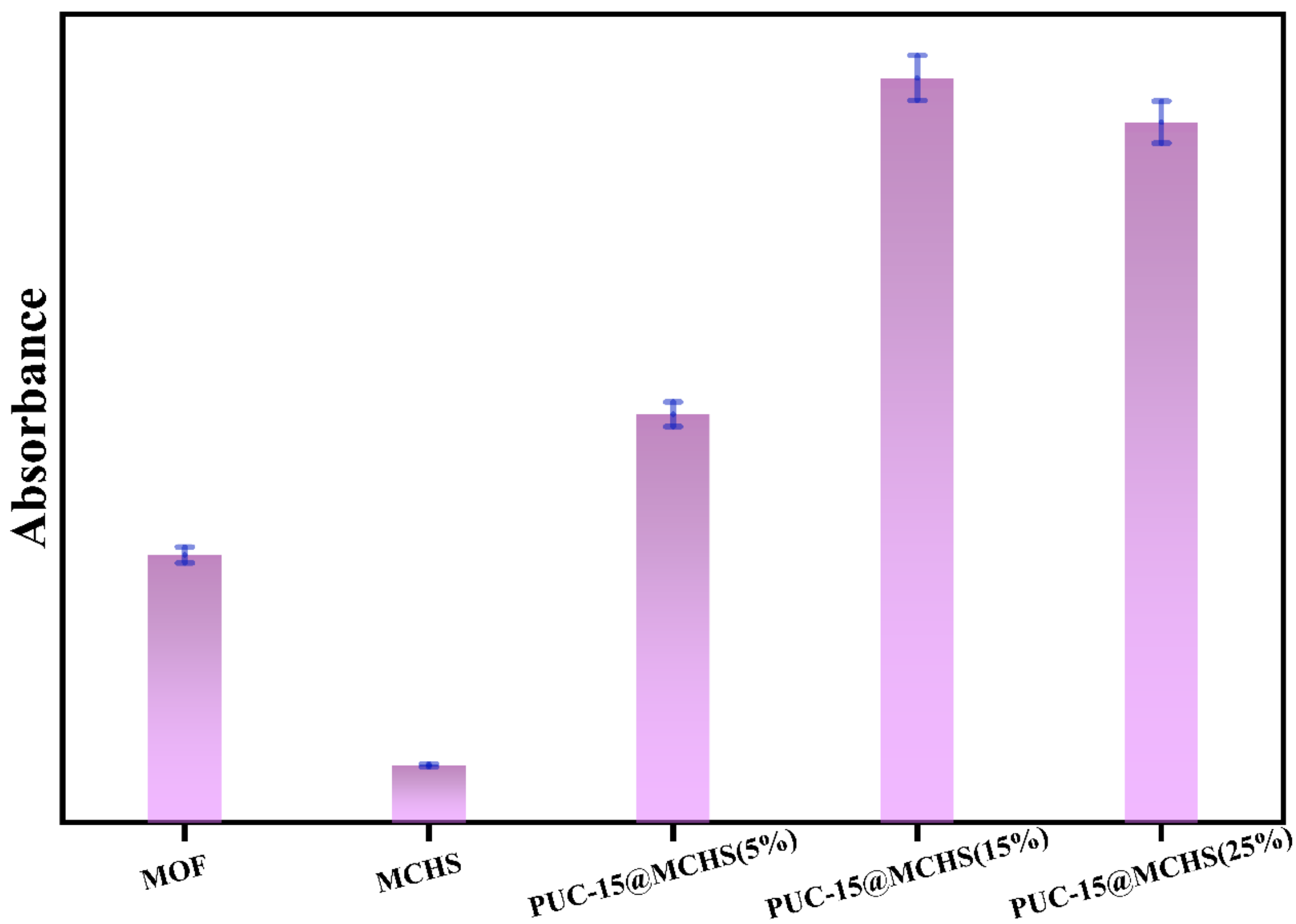

Figure S16. Comparative catalytic activity of MOF, MCHS, and PUC-15@MCHS composites with varying compositions.

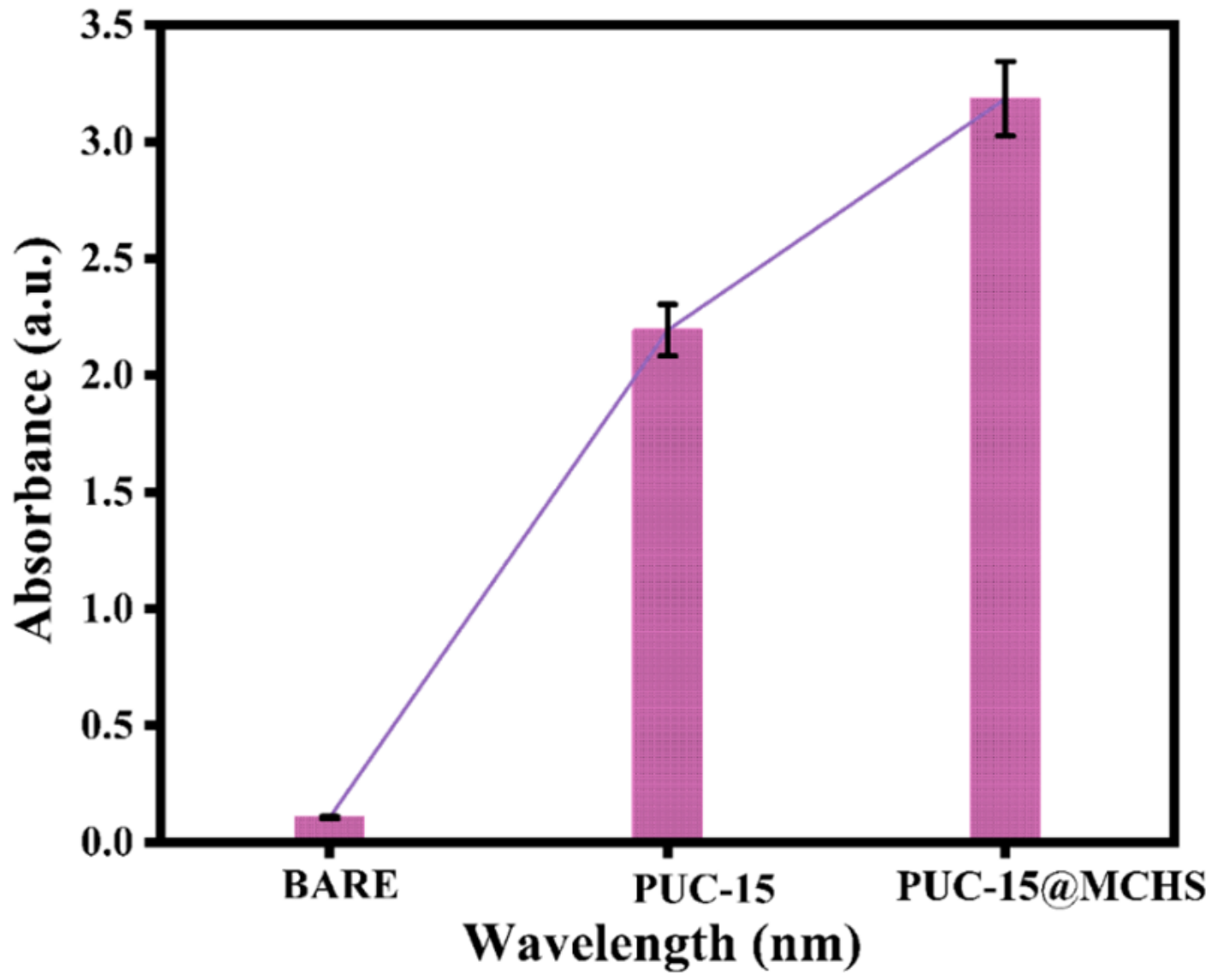

Figure S17. Bar graph illustrating the comparative catalytic activity of different materials: (1) Bare system (without catalyst), (2) pristine MOF (PUC-15), and (3) composite (PUC-15@MCHS). The composite exhibits significantly enhanced peroxidase-like activity, highlighting the synergistic effect of MCHS integration with the MOF structure.

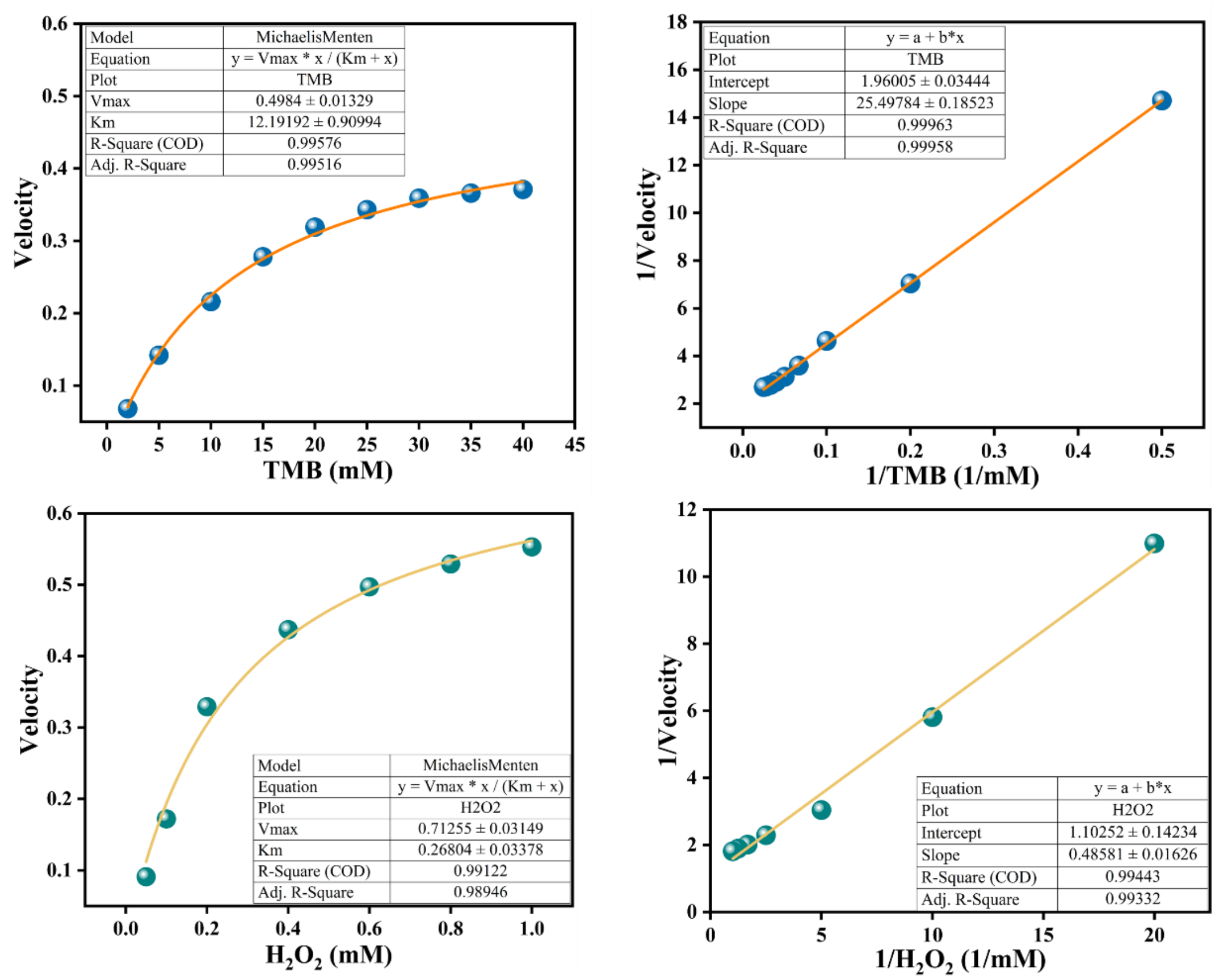

Figure S18. Steady-state kinetic analysis of pristine PUC-15 toward TMB oxidation in the presence of H_2O_2.

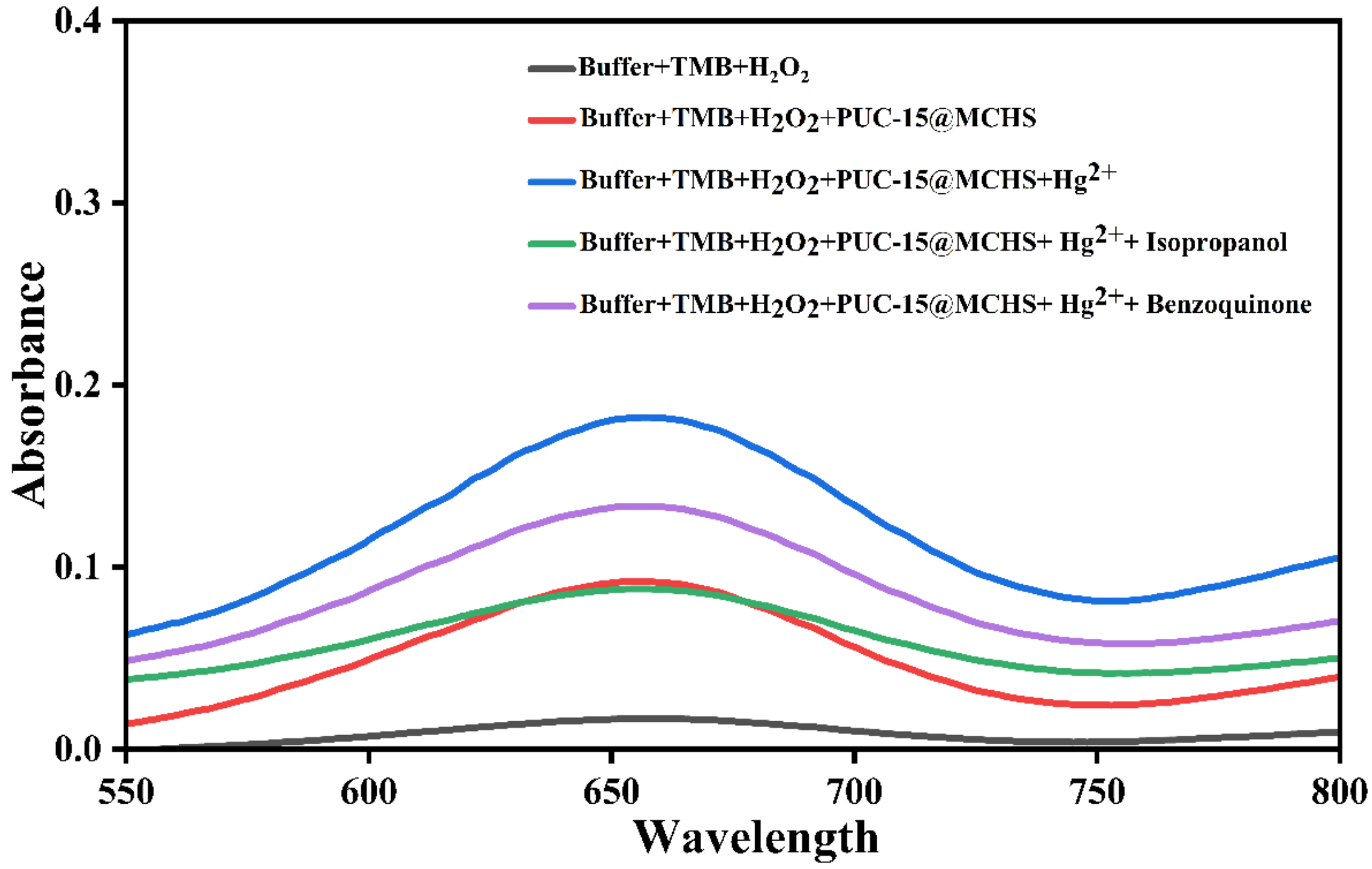

Figure S19. UV-Vis absorption spectra of the TMB/H_2O_2 system in the presence of PUC-15@MCHS, Hg^{2+}, and radical scavengers (isopropanol and benzoquinone), demonstrating the involvement of reactive oxygen species in the catalytic process.

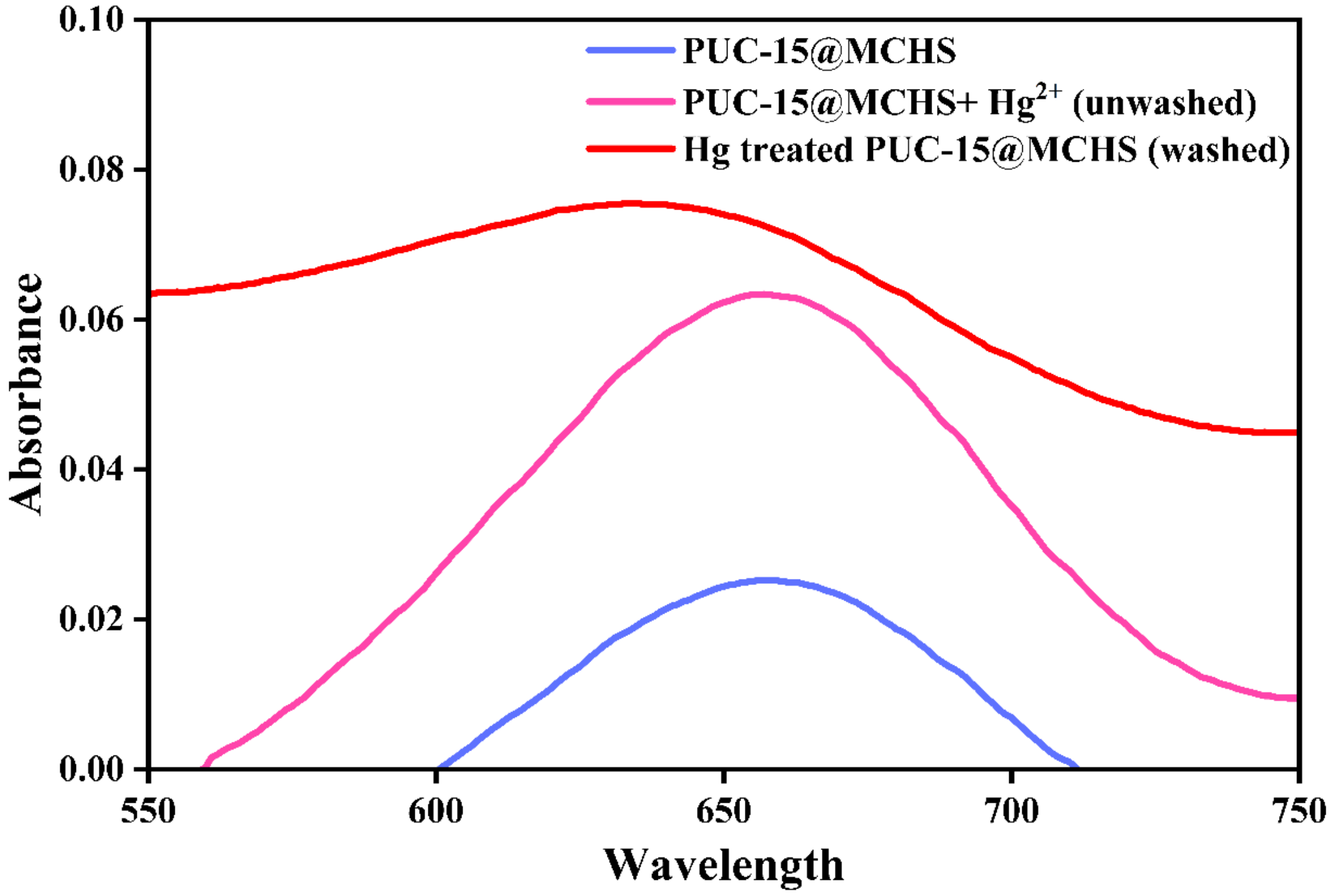

Figure S20. Comparative absorption spectra of PUC-15@MCHS, PUC-15@MCHS + Hg^{2+} (unwashed), and Hg-treated PUC-15@MCHS (washed), indicating the effect of Hg^{2+} interaction on the optical behavior of the composite.

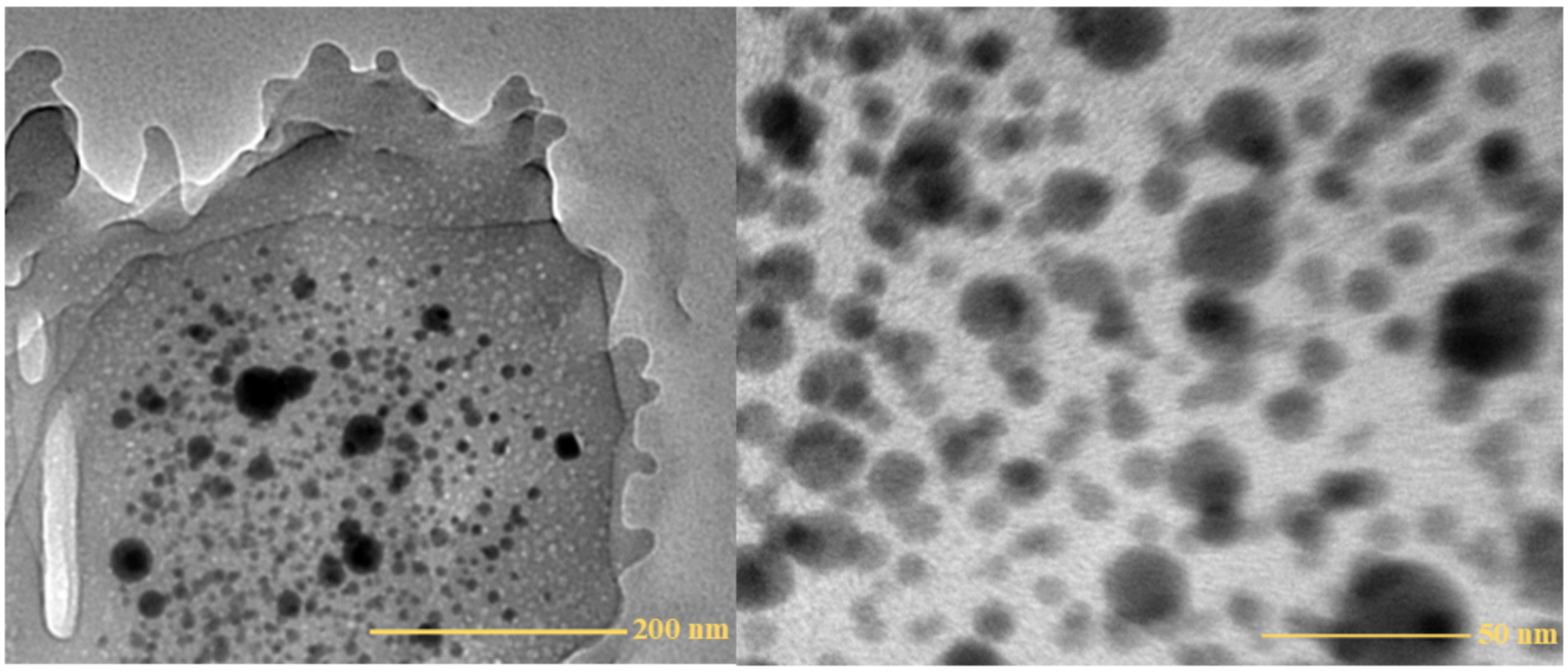

Figure S21. Hr-TEM images of the Hg-treated PUC-15@MCHS.

Table S1. Selected bond lengths (Å) of PUC-15

Zn(1)-N(1)	2.077(2)	O(2)-C(1)	1.230(3)
Zn(1)-O(1)	1.9547(19)	O(1)-C(1)	1.271(3)
Zn(1)-O(3)	1.9710(18)	O(3)-C(8)	1.266(3)
Zn(1)-O(5)	1.969(2)	O(4)-C(8)	1.236(3)
N(2)-C(13)	1.396(4)	O(7)-C(13)	1.196(3)
N(2)-C(9)	1.403(4)	O(8)-C(9)	1.202(3)
N(2)-C(6)	1.459(3)	O(5)-C(22)	1.213(4)
N(1)-C(16)	1.330(3)	O(6)-C(22)	1.240(4)
N(1)-C(20)	1.336(3)	C(22)-C(23)	1.488(5)

Table S2. Selected Bond Angles (°) of PUC-15

O(1)-Zn(1)-O(5)	105.73(9)	O(2)-C(1)-C(2)	119.6(3)
O(1)-Zn(1)-O(3)	124.26(8)	O(1)-C(1)-C(2)	116.1(3)
O(5)-Zn(1)-O(3)	113.44(9)	O(4)-C(8)-O(3)	124.2(3)
O(1)-Zn(1)-N(1)	113.10(8)	O(4)-C(8)-C(4)	118.3(3)
O(5)-Zn(1)-N(1)	94.35(10)	O(3)-C(8)-C(4)	117.5(3)
O(3)-Zn(1)-N(1)	102.01(8)	O(8)-C(9)-N(2)	120.8(3)
C(13)-N(2)-C(9)	126.2(2)	O(8)-C(9)-C(10)	123.7(3)
C(13)-N(2)-C(6)	115.0(2)	N(2)-C(9)-C(10)	115.5(3)
C(9)-N(2)-C(6)	118.8(2)	O(7)-C(13)-N(2)	120.1(3)
C(16)-N(1)-C(20)	116.1(2)	O(7)-C(13)-C(12)	123.2(3)
C(16)-N(1)-Zn(1)	120.77(18)	N(2)-C(13)-C(12)	116.7(3)
C(20)-N(1)-Zn(1)	123.1(2)	N(1)-C(16)-C(17)	123.5(3)
C(1)-O(1)-Zn(1)	115.99(19)	N(1)-C(20)-C(19)	123.3(3)
C(8)-O(3)-Zn(1)	116.65(18)	O(5)-C(22)-O(6)	122.2(4)
C(22)-O(5)-Zn(1)	116.6(3)	O(5)-C(22)-C(23)	120.1(4)
O(2)-C(1)-O(1)	124.2(3)	O(6)-C(22)-C(23)	117.6(4)

Table S3. Elemental composition of MCHS, pristine PUC-15, and PUC-15@MCHS composites.

**Sample**	**C (%)**	**N (%)**	**O (%)**	**Zn (%)**
MCHS	100	-	-	-
PUC-15 (MOF)	81.58	7.82	6.8	1.37
PUC-15@MCHS-5%	58.47	6.53	31.09	3.92
PUC-15@MCHS-15%	64.84	7.92	23.29	3.95
PUC-15@MCHS-25%	88.26	7.2	3.08	0.53

Table S4. XPS binding energy shift comparison of C 1s, N 1s, O 1s, Zn $2p_{1/2}$ and Zn $2p_{3/2}$ for the MOF and composite.

**XPS Peak**	**PUC-15 (eV)**	**PUC-15@MCHS (eV)**	**Binding Energy Shift (eV)**
**C-C/C=C**	**285.04 eV**	**285.20 eV**	**+O.16**
**O-C/C=O**	**288.30 eV**	**288.41 eV**	**+0.11**
**N1s**	**400.35 eV**	**400.15 eV**	**-0.20**
**O1s**	**531.62 eV**	**533.02 eV**	**+1.40**
**Zn $2p_{1/2}$**	**1045.2 eV**	**1045.02 eV**	**-0.18**
**Zn $2p_{3/2}$**	**1021.8 eV**	**1022.14 eV**	**+0.34**

Table S5. Comparative analysis of PUC-15@MCHS with reported Hg^{2+} sensors.

Serial No.	Sensor material	Method	Detection limit	Reference
**1.**	N,S-CDs	Fluorescence	5.4 µM	1
**2.**	L-Cys/CuNCs@ESM	Fluorescence	0.52 µM	2
**3.**	Single-atom Cu–C–N nanozyme	Colorimetry	50 nM	3
**4.**	Cu NCs	Fluorescence	33 µM	4
**5.**	T-CuNCs	Colorimetry	11.67 mM	5
**6.**	Nitrogen-carbon dots	Fluorescence	0.65 µM	6
**7.**	AgNPs	Electrochemical	8.43 µM	7
**8.**	PVP-AgNPs	Colorimetry	10 µgL^{-1}	8
**9.**	(E)-4-((4-(dimethylamino)phenyl)-diazenyl)-N(2mercaptophenyl)-benzenesulfonamide	Colorimetry	80 µM	9
**10.**	Ag NPs	Colorimetry	2.2 µM	10
**11.**	Au@AgPt NPs	Colorimetry	0.52 µM	11
**12.**	Azobenezene derivatives (Naked Eye)	Colorimetry	20 µM	12
**13.**	terpyridine deq/rivatives (Naked Eye)	Colorimetry	25 µM	13
**14.**	*p*-phenylenediamine functionalized AgNPs	Colorimetry	0.80 µM	14
**15.**	PUC-15@MCHS	Colorimetric-Nanozymatic	0.59 µM	**This Work**

Table S6. Comparison of Hg^{2+} sensors based on practical performance parameters.

Sensor/Material	Sensing Medium	pH Working Range	Response Time	Selectivity (ions tested)	Ref.
Porphyrinic MOF (LMOF, dual-mode fluorescence/colorimetric)	Aqueous	Not specified	Approximately 2 min	Good	15
DNA@MOF hybrid biosensor (Cu-MOF + PMDNAzyme)	PBS buffer (pH 7.4, fixed)	Buffer-fixed at pH 7.4	5 min (incubation)+ pre-treatment	Good	16
Rhodamine B/6G hydrazide probes (RBH/R6GH)	10% v/v MeOH/H_2O	Not reported	10-30 min	Good	17
Rhodamine B hydrazide chemosensor (MTRH, turn-on)	Aqueous	pH 7.4 only (neutral-fixed)	15 min	Good	18
Dual-mode biosensor (CuNCs@Zr-MOF + G-quadruplex DNAzyme)	PBS buffer (pH 7.4, fixed)	Narrow, buffer-fixed	5 min	Moderate	18
Eu-Ca-MOF ratiometric Ln-MOF sensor	Aqueous	Not specified	Not reported (equilibration required)	Moderate	20
Eosin Y-based chemosensors (EY-SO/EY-SM, turn-on)	Aqueous, neutral-basic only	pH 6–9 (basic-biased)	5–10 min	Good	21
Thiophene–-chiff base (PMP)	Aqueous/DMSO co-solvent	Moderate	Several minutes	Good	22
N-heteroaromatic small-molecule optical sensors	Organic co-solvent required	Narrow, pH-sensitive	Variable (minutes)	Variable across reports	23
Pillar[5]arene supramolecular fluorescent system	100% water	Not specified	Not reported	Moderate	24
**Present work**	**Aqueous acetate buffer (no co-solvent)**	**pH 2.0–5.5 (optimal pH 3.0)**	**10 seconds (instantaneos)**	**Excellent (15competing ions)**	**This work**

Table S7. Literature comparison of K_m values for TMB and H_2O_2 for natural horseradish peroxidase and selected peroxidase-like nanozyme catalysts.

Catalyst	K_m(TMB, mM)	K_m(H_2O_2, mM)	Reference
HRP (natural enzyme)	0.43	3.7	25
Fe3O4@ZIF-8 nanozyme	0.39	0.23	26
CuCeTA nanoflowers	0.54	0.62	27
Ag@bpp nanozyme	3.95	4.68	28
Fe-N-C single-atom nanozyme	3.6	12.2	29

Cu-MOF nanoparticles	47.7	2646	30
PUC-15@MCHS	9.88	0.27	This Work

Table S8. Real Water Sample Analysis for Hg^{2+} Detection Using PUC-15@MCHS Nanozyme Composite. (DW = Distilled water; CTW = Tap water from Chandigarh, RW = Gagghar River water, GW = Groundwater, and IPW = Industrial pharmaceutical water).

**Sample name**	**Spiked Concentration (µM)**	**Found Concentration (µM)**	**Recovery (%)**	**RSD (±)**
DW	0.00	0.01	100.0%	0.9
	1.00	0.92	92.0%	4.5
	5.00	4.89	97.8%	2.8
	10.00	9.67	96.7%	2.1
CTW	0.00	0.04	100.0%	0.8
	1.00	0.96	96.0%	3.9
	5.00	5.21	104.2%	3.2
	10.00	10.28	102.8%	2.4
RW	0.00	0.06	100.0%	0.9
	1.00	0.88	88.0%	5.2
	5.00	4.73	94.6%	4.1
	10.00	9.45	94.5%	3.3
GW	0.00	0.05	100.0%	0.8
	1.00	0.91	91.0%	4.7
	5.00	5.18	103.6%	3.4
	10.00	10.35	103.5%	2.6
IPW	0.00	0.03	100.0%	0.9
	1.00	0.94	94.0%	4.1
	5.00	4.95	99.0%	2.9
	10.00	9.82	98.2%	2.3

References

1 M. Pajewska-Szmyt, B. Buszewski and R. Gadzała-Kopciuch, *Mater. Chem. Phys.*, 2019,122484.

2 C. Zhang, M. Liang, C. Shao, Z. Li, X. Cao, Y. Wang, Y. Wu and S. Lu, *ACS Appl. Bio Mater.*, 2023, **6**, 1283–1293.

3 J. Ge, Y. Yuan, H. Yang, R. Deng, Z. Li and Y. Yang, *Mater. Today Chem.*, 2024, **37**, 102037.

4 M. Jiao, Y. Li, Y. Jia, L. Xu, G. Xu, Y. Guo and X. Luo, *Microchim. Acta*.

5 S. Li, Z. Zeng, C. Zhao, H. Wang, X. Ye and T. Qing, *New J. Chem.*, 2022, **46**, 4687–4692.

6 G. Ren, Y. Meng, Q. Zhang, M. Tang, B. Zhu, F. Chai, C. Wang and Z. Su, *New J. Chem.*, 2018, **42**, 6824–6830.

7 E. Eksin, A. Erdem, T. Fafal and B. Kıvçak, *Electroanalysis*, 2019, **31**, 1075–1082.

8 Monisha, K. Shrivas, T. Kant, S. Patel, R. Devi, N. S. Dahariya, S. Pervez, M. K. Deb, M. K. Rai and J. Rai, *J. Hazard. Mater.*, 125440.

9 R. Kaushik, A. Singh, A. Ghosh and D. A. Jose, *ChemistrySelect*, 2016, **1**, 1533–1540.

10 K. Farhadi, M. Forough, R. Molaei, S. Hajizadeh and A. Rafipour, *Sensors Actuators, B Chem.*, 2012, **161**, 880–885.

11 C. Song, J. Li, Y. Sun, X. Jiang, J. Zhang, C. Dong and L. Wang, *Sensors Actuators, B Chem.*, 2020, **310**, 127849.

12 X. Cheng, Q. Li, C. Li, J. Qin and Z. Li, *Chem. - A Eur. J.*, 2011, **17**, 7276–7281.

13 R. Shunmugam, G. J. Gabriel, C. E. Smith, K. A. Aamer and G. N. Tew, *Chem. - A Eur. J.*, 2008, **14**, 3904–3907.

14 S. Bothra, J. N. Solanki and S. K. Sahoo, *Sensors Actuators, B Chem.*, 2013, **188**, 937–943.

15 J. Yang, Z. Wang, Y. Li, Q. Zhuang, W. Zhao and J. Gu, *RSC Adv.*, 2016, **6**, 69807.

16 N.-H. Huang, R.-T. Li, C. Fan, K.-Y. Wu, Z. Zhang and J.-X. Chen, *J. Inorg. Biochem.*, 2019, **197**, 110690.

17 S. Kraithong, P. Damrongsak, K. Suwatpipat, J. Sirirak, P. Swanglap and N. Wanichacheva, *RSC Adv.*, 2016, **6**, 10081–10090.

18 J. Chen, J. Tao, H.-F. Yu, C.-P. Ma, F. Tan and X.-C. Wang, *Spectrochim. Acta, Part A*, 2023, **296**, 122648.

19 S. Jain, M. Nehra, N. Dilbaghi, G. R. Chaudhary and S. Kumar, *BME Front.*, 2024, **5**, 0078.

20 N. Wu, H. Guo, L. Peng, Y. Chen, Y. Liu, C. Li, H. Zhang and W. Yang, *Talanta*, 2022, **250**, 123710.

21 B. Kumar, V. Muthukumar, A. Chakraborty, A. Mahato, B. Ghosh and N. Das, *Mater. Adv.*, 2026, **7**, 4596–4610.

22 P. Sunitha, B. G. Gowda, P. Singh, G. Vengatesh, K. Varadaraju, K. S. Abass, V. Stupin, E. Silina and S. P. Kollur, *Sci. Rep.*, 2025, **15**, 33207.

23 K. Debsharma, S. Dey, B. Dutta and S. Dey, *Analyst*, 2025, **150**, 2725–2775.

24 X. Jiang, L. Wang, X. Ran, H. Tang and D. Cao, *Biosensors*, 2022, **12**, 571.

25 L. Gao, J. Zhuang, L. Nie, J. Zhang, Y. Zhang, N. Gu, T. H. Wang, J. Feng, D. Yang, S. Perrett and X. Yan, *Nat. Nanotechnol.*, 2007, **2**, 577–583.

26 D. Chen, Y. Li, S. Xu, Z. Guo, Q. Yao and X. Chen, *Microchem. J.*, 2024, **200**, 110449.

27 C. Jiang, X. Xie, Y. Luo, S. Yu, H. Peng, X. Hou and C. Zheng, *J. Mater. Chem. B*, 2023, **11**, 9630–9638.

28 S. Ullah, M. Asad, F. Ullah, M. Shah, K. F. Fawy, X. Zhang, W. Sun, U. Nishan and A. Badshah, *RSC Adv.*, 2025, **15**, 49288–49300.

29 L. Jiao, W. Xu, H. Yan, Y. Wu, C. Liu, D. Du, Y. Lin and C. Zhu, *Anal. Chem.*, 2019, **91**, 11994–11999.

30 S. Wang, W. Deng, L. Yang, Y. Tan, Q. Xie and S. Yao, *ACS Appl. Mater. Interfaces*, 2017, **9**, 24440–24445
